# Monitoring Methylmalonic Aciduria by NMR Urinomics

**DOI:** 10.3390/molecules25225312

**Published:** 2020-11-14

**Authors:** Alina Nicolescu, Daniela Blanita, Chiril Boiciuc, Victoria Hlistun, Mihaela Cristea, Dorina Rotaru, Ludmila Pinzari, Ana Oglinda, Adela Stamati, Isabela Tarcomnicu, Andreea Tutulan-Cunita, Danae Stambouli, Sergiu Gladun, Ninel Revenco, Natalia Uşurelu, Calin Deleanu

**Affiliations:** 1 “Petru Poni” Institute of Macromolecular Chemistry, Romanian Academy, Aleea Grigore Ghica Voda 41A, RO-700487 Iasi, Romania; ciobanu.mihaela20@yahoo.com; 2 “C. D. Nenitescu” Centre of Organic Chemistry, Romanian Academy, Spl. Independentei 202B, RO-060023 Bucharest, Romania; 3 Institute of Mother and Child, Str. Burebista 93, MD-2062 Chisinau, Moldova; blanita.daniela@gmail.com (D.B.); tervenom@gmail.com (C.B.); hlistunvica@yahoo.com (V.H.); liudmilapinzari@rambler.ru (L.P.); gladunsergiu@gmail.com (S.G.); 4 “Gheorghe Palade” City Clinical Hospital, Str. Melestiu 20, MD-2001 Chisinau, Moldova; dorinarotaru@rambler.ru; 5 “Nicolae Testemitanu” State University of Medicine and Pharmacy, Bd. Stefan cel Mare si Sfint 165, MD-2004 Chisinau, Moldova; ana.oglinda@usmf.md (A.O.); adela.stamati@usmf.md (A.S.); ninel.revenco@usmf.md (N.R.); 6 Cytogenomic Medical Laboratory, Calea Floreasca 35, RO-014453 Bucharest, Romania; isa.tarcomnicu@gmail.com (I.T.); cunita@cytogenomic.ro (A.T.-C.); stambouli@cytogenomic.ro (D.S.)

**Keywords:** methylmalonic aciduria, methylmalonic acid, NMR spectroscopy, urine, metabolomics

## Abstract

The paper reports on monitoring methylmalonic aciduria (MMA)-specific and non-specific metabolites via NMR urinomics. Five patients have been monitored over periods of time; things involved were diet, medication and occasional episodes of failing to comply with prescribed diets. An extended dataset of targeted metabolites is presented, and correlations with the type of MMA are underlined. A survey of previous NMR studies on MMA is also presented.

## 1. Introduction

The first demonstration of potential use of nuclear magnetic resonance (NMR) spectroscopy in analyzing blood plasma was published by J. K. Nicholson and P. J. Sadler in the mid-1980s once the 400 MHz NMR instruments widely penetrated the chemical community [1]. Shortly after, that the same group carried out NMR pioneering works in urine analysis [2,3,4,5,6]. In spite of the great number of papers published on NMR urine analysis, and of the existence of several groups around the world active in the field, there are only a few published results on metabolite ranges determined by NMR. Many of these data are being kept as in-house raw databases. Thus, most of the normal ranges for various metabolite concentrations published to date where obtained by classical methods [7,8,9]. An early reference for normal values for metabolite concentrations in urine obtained by NMR has been published by the Zuppi’s group [10,11,12]. The same group also described the comparison of metabolite concentrations for control populations from two different geographical regions [13]. We also published metabolite concentrations for normal and diabetes groups from Eastern Europe (Bucharest), and we discussed the results in comparison with previously published data [14,15]—our interests covering diagnoses of various metabolic disorders [16,17,18,19].

Metabolomics involves simultaneous detection of a large number of compounds from a living system. Although this goal was achieved earlier by using a panel of classical clinical laboratory techniques, the number of metabolites was limited in early days to those considered as direct markers for a disease. The control and pathological groups have been also limited in general to the minimum necessary for developing and validating clinical diagnosis tests. It was only when MS techniques started to be widely employed in the clinical environment that metabolomics became available as a screening test. Currently there are only two analytical techniques, namely, MS and NMR spectroscopy, which are suitable for large metabolomics screenings of various pathologies and for epidemiological studies. Each of these techniques has several advantages and drawbacks in comparison with the other, and ideally they are used as complementary techniques.

The main advantages of the NMR method are that it provides direct quantitative information on selected markers (even in the absence of a standard), that it provides an untargeted global biochemical profile, and that it requires minimum sample preparation. Current state-of-the art NMR instruments are sufficiently stable and provide highly reproducible results, allowing the possibility of using NMR spectra acquired by different operators, on different instruments, in different countries in the same statistical model, which a few years ago was only a dream. Thus, NMR instrumentation provides unsurpassed capabilities in terms of reproducibility and speed, being suitable for both diagnoses and long-term monitoring of metabolic disorders.

When evaluating metabolic disorders, the NMR approaches rely on two somewhat different methods, namely, targeted and non-targeted metabolomics. The first one, often considered as “old fashioned” involves calculations of concentrations for specific metabolites based on well-assigned signals, whereas the later, often seen as the “modern” approach, looks to the whole NMR profile and classifies this pattern in order to predict medical diagnosis. In fact, the later approach does not exclude the additional calculation of targeted concentrations, and in our opinion both approaches should be used together in all cases when this is possible. Moreover, in order to provide information which can be cross-referenced against classical clinical textbooks by clinical practitioners, it is essential to provide quantitative results for targeted metabolites, even if this approach is labor intensive, requiring highly trained personnel, and slower, requiring more than double the instrument time. Whereas the number of possible targeted markers is limited and intrinsically lower in information in comparison to the untargeted global spectral profile, in the current state of transition from regular diagnosis and treatment to personalized diagnosis and treatment, in our opinion it is mandatory to provide both types of results, i.e., targeted concentrations and non-targeted statistical discrimination. Whenever these complementary types of results are presented together, they contribute to checking and self-regulating the statistical models and further increase the confidence of non-NMR experts in adopting untargeted diagnosis approaches in clinical practice. As AI and big data are evolving very fast, blind diagnoses based on statistical models might become the standard approach in the not-so-distant future. We have previously discussed both targeted and non-targeted metabolomics in connection with diabetes, which is the most common metabolic disorder [14,15,20].

Rare diseases are currently considered those conditions affecting less than 1 in 2000 people and there are several such diseases affecting less than 1 in 100,000, and even less than 1 in 1,000,000 newborns [21,22,23]. To date there are over 6000 known rare diseases [23], and it was proven that the incidence of such diseases is variable from one geographical population to another [24]. Currently there are only a limited number of rare diseases which are screened at national scale and this number differs from country to country [25,26,27]. However, with the advent of MS and NMR instrumentation, in the current stage of 21st century with emerging personalized medicine and diagnosis, there is no excuse for developed countries to withhold from introducing large scale national screenings for all diseases detectable by MS and NMR techniques. It is likely that in the following few years the pressure from the general public will add to the already existing lobby of various patients associations, leading to such wider screenings which will constitute not only a huge benefit for the directly affected people, but also a gold mine for many types of epidemiological studies, including the evaluation of the extent of the spread of various pollutants and the effects of various environmental factors, food formulas, and lifestyle habits.

Hereditary metabolic disorders are caused by inborn errors of metabolism (IEM) due to mutations in genes which encode enzymes and proteins involved in cell metabolism, altering the normal metabolic pathways. In most cases the altered metabolic pathways lead to accumulations of toxic compounds in the body, and if not discovered and treated in early stages of life they result in irreversible mental and physical disabilities, in most cases being life threatening.

Methylmalonic aciduria is an inherited multifunctional autosomal recessive metabolic disorder belonging to the wider class of organic acidurias. Methylmalonic aciduria (MMA) is determined by a defect in one of the genes causing methylmalonic acid (MMAc) accumulation in body fluids, and it has the overall incidence estimated at 1:50,000 [9]. In over 60% of cases, MMA is caused either by mutations in the MMUT gene (methylmalonyl-CoA mutase) responsible for a missing or defective enzyme with the same name (methylmalonyl-CoA mutase) or by mutations in the MMAA gene (metabolism of cobalamin associated A) responsible for defective transport or synthesis of adenosyl-cobalamin (the cofactor of methylmalonyl-CoA mutase). MMA causes accumulation of acylcarnitine in blood and methylmalonic acid in urine. The excess of methylmalonic acid in the organism further leads to inhibition of succinate dehydrogenase, which is an enzyme involved in mitochondrial aerobic oxidation of glucose. Thus, MMA patients exhibit neurological and gastrointestinal problems with common symptoms such as lethargy, seizure, and anorexia [28]. Usually, the appearance of first clinical symptoms ranges from hours to weeks after birth, although in about 25% of cases the first symptoms appear after one year or even in adolescence or adulthood [9]. Moreover, some completely asymptomatic MMA cases have been also reported [29,30]. The effects of MMA vary from mild to life-threatening and its treatment is complex, requiring regular monitoring and frequent therapeutic and dietary adjustments [19].

NMR, even from its early stages of application in medicine, has proven its potential in diagnosing MMA. Thus, the first papers describing the detection of methylmalonic acid in urine of MMA patients came from the group of R.A. Iles [31,32]. Further studies detailed the NMR diagnosis of MMA based on the presence of methylmalonic acid in urine [33,34], demonstrating also the good correlation between NMR and MS for the metabolite/creatinine (Crn) ratios [35]. An early paper was clearly describing the selectivity of NMR in differentiating between methylmalonic aciduria (MMA) and propionic aciduria (PA) [36], the two most common disorders caused by failures in the propionate metabolism [37]. Over the years, NMR proven its utility in the study of other body fluids of MMA patients. Thus, vitamin B12 deficiency was associated by ^1^H-NMR spectroscopy with the MMAc concentrations in cerebrospinal fluid (CSF) [38], and localized ^1^H-NMR (MRS) studies associated changes in *N*-acetylaspartate (NAA), lactate (Lac), creatine (Crea), and choline (Cho) in the brain regions of MMA patients [39,40].

## 2. Results and Discussion

The first MMA case was reported by Oberholzer in 1967 in an infant with ketoacidosis and large amounts of methylmalonic acid in the blood and urine [41]. The disorder has been later assessed as being caused either by a deficiency in methylmalonyl-CoA mutase, or by the defective synthesis of adenosylcobalamin [42,43]. To date, the only unambiguous diagnoses of MMA are based on MS, NMR spectroscopy, or genetic tests. All the other fast tests and/or symptoms evaluation alone can only narrow the first diagnostic as an organic aciduria, and out of these collective disorders only maple syrup urine disease (MSUD) may be unambiguously assigned based on simple chromatography of plasma aminoacids [9].

Although there are several papers reporting on diagnosis and ranges of the main MMA marker in urine [44,45,46], there is a scarcely of reports on variations of MMA metabolites in the same individual over a longer period of time and not only as a “one-shot” in the statistical cohort. Additionally, the concentration ranges of non-specific MMA markers have not been reported in MMA patients. Thus, due to limitations in the number of available samples, most of the studies are limited to descriptive analysis. Unlike such studies, we report on a wide range of metabolite concentrations which could serve as background for clinical diagnosis, therapy response, and long term monitoring of MMA patients.

For this study the samples have been collected both during hospital visits and at home by parents at various times during the day, with the only constraint being rapid freezing. The collection method was on purpose; it allowed the convenience of the parents in order to evaluate the “natural” behavior and variations of the diseased child in everyday routines. Such an extensive evaluation of the child’s behavior could only be achieved by the NMR technique allowing simultaneous multiparameter evaluations on large numbers of samples. By contrast, the previously published papers focused mainly on the evaluation of the treatment as a response on a few selected markers, under controlled intervals after carnitine administration. This previous clinically controlled approach is very useful in the initial assessment of the response of a particular organism to the treatment, and for the establishment of the long-term therapeutic scheme. However, there is a gap in assessing the long term evolution of the organism under daily routine, including during occasional failures to comply with the therapeutic scheme, which often are not reported to the family doctor or physician in charge. The present paper is trying to fill this gap by providing the long-term, multiparameter monitoring of both specific and non-specific MMA markers in a non-clinical environment. Although in such “relaxed” collection conditions, the variations of many metabolites reported here would be higher than in a timely controlled trial, these variations should provide valuable information on how wide the real range of concentrations is, and if in some instances the extreme variations pose a life threatening risk.

The present study reports on five MMA cases involving 1 girl and 4 boys with ages ranging between 7 days and 11 years old, with the aim of filling the gap of simultaneous monitoring of multiple targeted metabolites.

**Figure 1:**
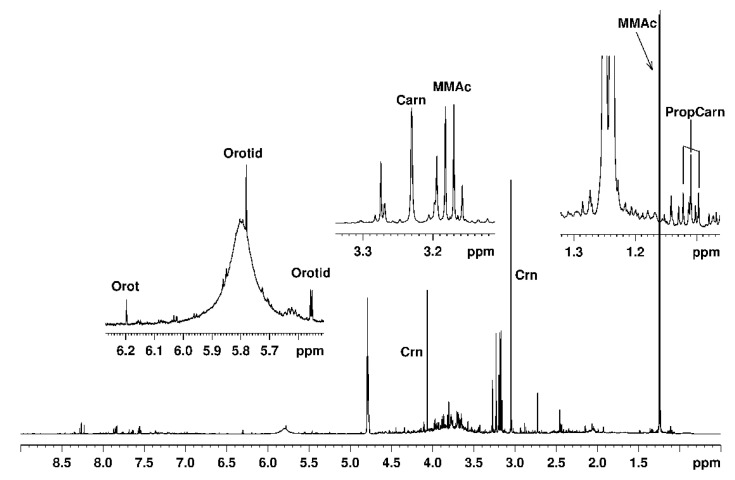
^1^H-NMR spectrum at 600 MHz of a urine sample from a 5½ y.o. boy with MMA (Case 5). Labeled signals exemplify assignments of metabolites relevant for the pathological condition.

Figure 1 shows the whole ^1^H-NMR spectrum with amplification of the relevant regions of a urine sample from a 5½ y.o. boy with MMA. The labeled signals exemplify markers for the NMR-based MMA diagnosis and quantitative monitoring in urine samples.

As in most of the previous reports the number of MMA patients was quite low (as expected for a rare disease), and as each paper deals with a very limited number of metabolites in addition to MMAc (not always the same additional metabolites), with results from patients with different ages and employing different techniques for metabolites quantitation, it is quite useful to try to provide in one shot as many metabolites concentrations for the same MMA patient as possible, even if at the current state of knowledge many of these metabolites are not connected with MMA. The cases reported in the present paper have been followed in detail by NMR over a long period of time, accounting thus for various situations in the same subject (organism), e.g., age evolution, diet, decompensation episodes, or metabolic crises, and one may easily compare our whole set of reported metabolites with normal values. Moreover, it is often the case that not only national regulations but individual laboratories have established their own reference values. We have been exemplifying earlier that when looking to various pathological metabolite values, one cannot use absolute reference concentrations from various geographical regions in the same model; instead, when comparing differences between controls and pathological groups they hold true across geographical regions and laboratories [14].

A compilation of reference ranges for urine by C. Lentner was very popular in the early stages of NMR and it is still quite useful today [47]. Since then, several compilations of reference ranges for urine metabolites have been published, with a very good book being published by N. Blau et al. [8]; and there are extensive Internet resources which are also worth being mentioned [48,49,50]. Valuable reference concentrations in urine for infants and children [51,52], and neonates and infants [24,51,53], have been recently published. In most cases ranges of normal concentrations previously published represent compilations of single samples (one shot) from a large number of control cases. Normal values of methylmalonic acid in urine have been reported for 20 samples to range between 0.58 and 3.56 mmol/mol Crn [54]. Normal ranges of methylmalonic acid in urine have been also reported to be influenced by diet [55,56]. We present below a compilation of NMR accessible metabolite concentrations from 180 samples belonging to a group of five MMA patients followed over a period of several months. As usually samples from suspected IEM cases arrive in NMR laboratories more or less occasionally, it is useful to assess the expected range of variations in the same individual organism in various situations, including diurnal cycle, diet, medication, etc.

Table 1 and Table 2 below show concentration ranges for several NMR-detected metabolites. Extended tables, including both MMA-specific and non-specific metabolites, are presented as supplementary material (Tables S1 and S2). Although several of these metabolites are not markers for MMA, we have reported previously that such non-specific markers may show differences in diseases groups in comparison with controls [14,15,20]. In addition to global averages and ranges for each case, averages and ranges for periods with and without diets/treatments are also detailed in below tables.

The first observation from the data presented above is that the disease related markers (MMAc, orotic acid, orotidine, carnitine, and propionylcarnitine) show large variations in the same individual, depending on the treatment and general status of the child, this being in accordance with other previously monitored MMA cases. In addition to this, several other metabolites, such as valine, lactate, 3-hydroxyisovaleriate, and glycine, which are not MMA-specific markers, are showing deviations from controls.

**Figure 2:**
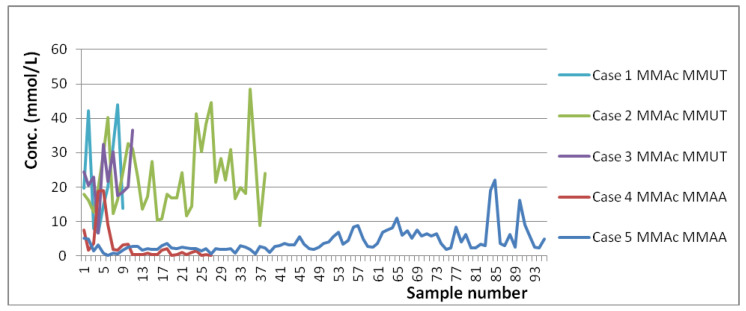
Absolute MMAc concentrations (mmol/L).

**Figure 3:**
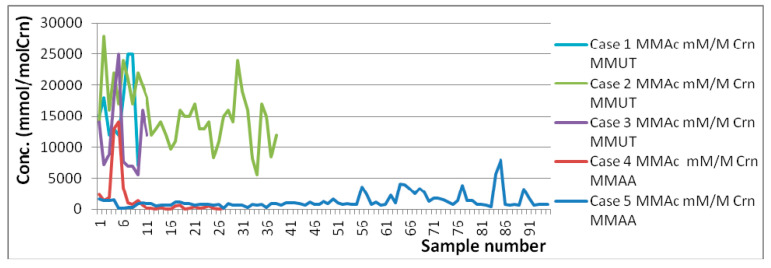
MMAc relative to Crn concentrations (mmol/mol Crn).

**Figure 4:**
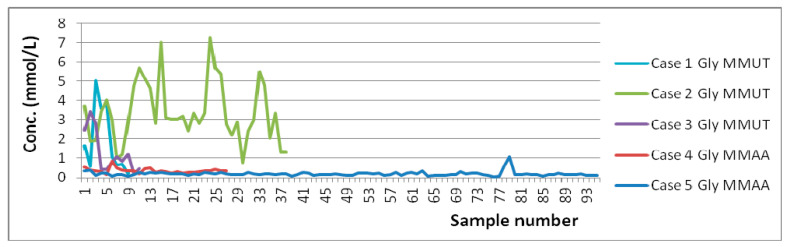
Absolute Gly concentrations (mmol/L).

In terms of MMAc ranges, one can see from the below graphs (Figure 2 and Figure 3) that in both MMAA mutation cases the concentration is in general kept below 10 mM, whereas in all three MMUT mutation cases the concentration is well above 10 mM in spite of the same type of treatment. One can also note that the few spikes in MMAA cases are from situations when the treatment was either interrupted or not started, showing, thus, a good response to treatment. However, even these MMAA spikes are quite low in comparison with MMUT average concentrations. Another metabolite which seems to strongly correlate with the type of mutation is glycine (Figure 4). From Table S2 (supplementary mater) one can also note that Ala in the case of MMUT has in general higher values than the ones reported in the literature for controls. Formate concentrations in the MMUT cases are also higher than those in the MMAA cases, although in all cases there are no significant deviations in concentration from previously reported controls.

**Figure 5:**
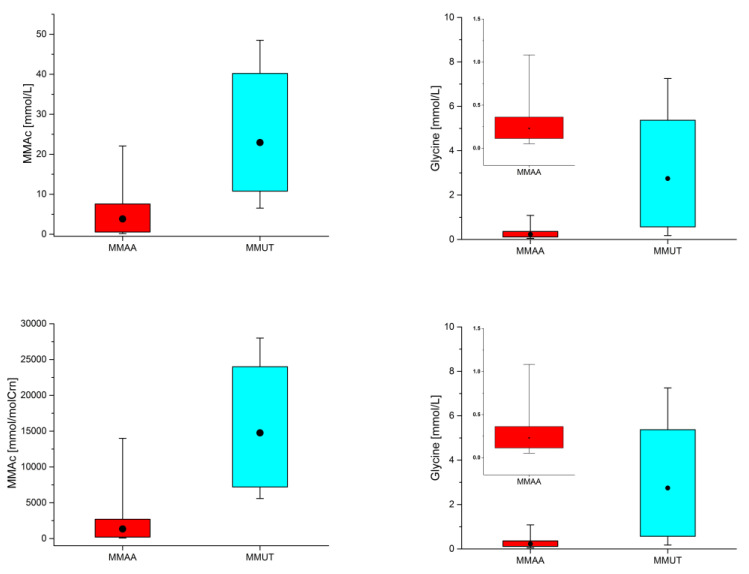
Overall combined data for all cases and time intervals. Upper graphs, absolute concentrations for MMAc and Gly; lower graphs, concentrations normalized to creatinine accounting for physiological dilution factor. Solid quadrilaterals represent 90% of all samples and solid dots represent the average concentrations.

When we consider the overall statistics for all data together, it becomes obvious (Figure 5) that both MMAc and Gly concentrations in MMA patients are mutation dependent. The level of separation of data allows even a pre-genetic NMR-based diagnosis of the mutation type with over 90% confidence.

As a direct response to therapy there are significant excretions of l-carnitine and propionylcarnite, and this is in accordance with previous reports discussed below. In terms of concentrations, in all MMAA cases Car was excreted in much higher amounts than in MMUT cases.

In one MMAA case (case 5), there were several amonemia episodes and we present the corresponding orotic acid and orotidine concentrations in the tables above. Such episodes are known to occur occasionally in MMA patients [60].

In terms of age ranges, it is important to stress that there is a good overlap of ages at least over some intervals, and this allows us confidently say that the observed differences in concentration ranges are explained by the disease (type of MMA mutation) and not by the age differences. This can be seen better from Figure 2, Figure 3 and Figure 4, wherein unlike Table 1 and Table 2, the evolutions of concentrations in time can be observed. Thus, due to the range of ages over which each case was monitored, it is obvious that there are moments when the same ages for various cases are present in the graphs from Figure 2, Figure 3 and Figure 4. In spite of both identical and different ages present in Figure 2, Figure 3 and Figure 4, the overall levels of concentration ranges remain well separated over the whole intervals of ages for the two mutations. Thus, one can see that, for instance, Case 3 monitored between 2½–3½ y.o. is well separated from Case 5 monitored between 2–11 y.o., and this is explained by the mutation type and not the age difference. As an additional general observation, the literature reported values for healthy individuals are more heterogenous in terms of age, covering neonates, infants, children, young, and old adults, whereas the overall age ranges for the five cases reported here cover mainly ages between 1 m.o. and 5 y.o., with only some samples collected at ages below 1 m.o. and over 5 y.o.

In addition to the presence of methylmalonic acid, previous publications indicated a few other abnormal metabolite levels in the urine of MMA patients. Thus, it was shown that the propionylcarnitine level in urine and blood increased with carnitine intake in MMA patients, indicating both a carnitine deficiency and a possible therapeutic effect [61,62,63]. NMR proved its monitoring power from the early stages of these findings [64].

An early NMR paper reported the MMA case of a 3 day old Japanese boy with failure to thrive, vomiting, loss of consciousness, and attacks of apnea. The clinical data reported severe metabolic acidosis, pH 6.55, ketonuria, hyperammonemia, and hyperglycinemia. In spite of the low quality of the published NMR spectrum in comparison with modern instruments, the authors clearly demonstrated after the 4th day until 19th day of life, there was a reduction of the methylmalonate concentration from 24,600 to 3500 mmol/mol Crn as a result of the 15 days of treatment with l-carnitine [36].

In the same year Iles reported the NMR monitoring of the effect of l-carnitine therapy on the excretion of several metabolites in a MMA child [65]. In the study a girl diagnosed with an apomutase which was not responsive to B12 therapy was maintained on oral therapeutic daily doses of l-carnitine from 21 months old (m.o.) and reassessed by NMR at 25 m.o. and 3 y.o. The paper described a severe metabolic crisis at the age of 25 months when 400 mg/kg l-carnitine was administrated over 32 h, resulting in a 50% reduction in excreted methylmalonic acid after 24 h from the initiation of the shock therapy, an increase in propionylcarnitine of up to 10,000 mmol/mol Crn and an increase in acetylcarnitine, and finally, an increase methylmalonic acid reaching 30,000 mmol/mol Crn after 66 h. The same patient was monitored by NMR at 7 y.o. before and after two intravenous doses of 100 mg/kg l-carnitine. Before the carnitine injection the methyl malonic acid level was 28,000 mmol/mol Crn; creatine (Crea) was 1000 mmol/mL Crn; and the pH was estimated as very low from the chemical shifts of methylmalonic acid, creatine, and creatinine. Eventually pH increased and propionylcarnitine and acetylcarnitine steadily increased, with propionylcarnitine reaching 16,000 mmol/mol Crn; and 40 h after the second carnitine injection, MMAc and Crea decreased to 8000 and 400 mmol/mol Crn respectively. In both episodes the clinical status improved in parallel with the metabolic changes observed in urine [65]. A similar trend for Crea/clinical status was reported by the same group in another MMA patient which was not subjected to carnitine therapy [34].

It is worth mentioning that when l-carnitine is administrated to healthy persons, it is excreted about 70% unchanged and 30% as acetylcarnitine [65].

Ando et al. reported methylcitric acid (MCAc) in the urine of MMA and PA patients for the first time [66]. MCAc is generated from accumulated propionyl-CoA by interrupting the citric acid cycle [67]. It was shown that from the four possible diastereomers of MCAc, only two of them, namely, 2*S*,3*S* and 2*R*,3*S*, are present in urine of MMA, PA, and holocarboxylase synthetase deficiency (HCSD) patients [68,69]. Krawczyk assigned for the first time the MCAc signals in the urine of a PA patient showing a (2*S*,3*S*):(2*R*,3*S*) ratio of 1:1.2 at pH 12.5 [70]. Karwczyk also quantified MCAc together with MMAc in two MMA patients. In order to unambiguously assign the MCAc signals, the ^1^H-NMR and HMQC spectra were run at pH 2.5 and 12.5. The concentrations at pH 2.5 for the first patient were 795 and 3591 mmol/mol Crn for MCAc and MMAc, respectively, and for the second patient 483 and 1449 mmol/mol Crn for MCAc and MMAc, respectively. The ratio of (2*S*,3*S*):(2*R*,3*S*) isomers was reported as 1.08:1 in the first case and 1:1.15 in the second case [71], which is somehow surprising, as one would expect similar ratios when the same biochemical pathway is involved.

In the presently reported cases we did not detect MCAc and this is in accordance with previous reports suggesting that the increased pH induced by carnitine may have as an additional positive outcome the prevention of the formation of MCAc from propionly-CoA and oxaloacetate [65].

Propionylcarnitine was mentioned as the best MS marker for both MMA and PA [37], and our current report supports this assessment with the additional note that there should be samples recorded from several excretion moments for each case in order to make sure that this metabolite is spotted.

The urine from two patients with MMA was monitored by ^1^H-NMR spectroscopy during a metronidazole administration clinical trial. Thus, the patients were monitored by NMR several days before and after the administration of metronidazole, and it was concluded that the propionylcarnitine excretion was not affected by metronidazole [72]. The MMA patients have been under good metabolic control and they have been receiving l-carnitine as a supplement. The NMR study concluded that in spite of a total propionate (TPr) metabolites’ reduction by 40% during metronidazole therapy, the propionylcarnitine (PrCar) level was unaffected. Thus, the first patient, a 3½ y.o., exhibited in 10 samples before metronidazole administration, 832 (571–1069) mmol PrCar/mol Crn, 11,620 (8500–16,338) mmol TPr/mol Crn, with PrCar representing 7% from TPr; and the seven samples after administration of metronidazole exhibited 824 (473–1125) mmol PrCar/mol Crn and 7750 (3345–12,162) mmol TPr/mol Crn, with PrCar increasing to 11% from TPr. The second patient, a 7 m.o., exhibited in 15 samples before metronidazole administration 50 (24–97) mmol PrCar/mol Crn and 811 (457–1350) mmol TPr/mol Crn, with PrCar representing 6% from TPr; and the 13 samples after administration of metronidazole exhibited 57 (28–143) mmol PrCar/mol Crn and 604 (214–1286) mmol TPr/mol Crn, with PrCar increasing to 9% from TPr. Metronidazole induced a reduction of MMAc by 30% in the first patient and by 20% in the second patient. Previous studies reported significant reduction of propionate levels by antibiotics such as neomycine [73] and metronidazole [74,75], but they did not consider propionylcarnitine separately.

In the present study we detected PrCar in all five MMA cases during periods when patients were subjected to carnitine (Car) administration (Table 1 and Table 2).

## 3. Materials and Methods

### 3.1. MMA Cases

All MMA patients have been enrolled with the Institute of Mother and Child for specific treatment and family counseling. The cases consisted of one boy (Case 1) monitored by NMR over a period of nine months between 7 days and 9 m.o., one boy (Case 2) monitored by NMR over a period of one and a half year, between 11 days and 1½ y.o., one boy (Case 3) monitored by NMR over a period of one year between 2½ and 3½ y.o., one girl (Case 4) monitored by NMR over a period of nine months between 4 years, 9 months and 5½ y.o., and one boy (Case 5) monitored by NMR over a period of nine years between 2 and 11 y.o. All cases have been diagnosed by specific clinical manifestation, tandem MS, and the MMA mutation types assigned by molecular genetics. All patients have been subjected to the same type of diet with reduced protein intake and oral administration of L-carnitine and B12 vitamin. The study was approved by the Ethics Committee of the Institute of Mother and Child, Chisinau, No. 01-13/1380 from 17.05.2017. All subjects gave their informed consent for inclusion in the study. The study was conducted in accordance with the Declaration of Helsinki.

### 3.2. Sample Collection

The urine samples were collected in sterile containers with tight-fitting covers. The urine samples were frozen and stored at −20 °C until the first ^1^H-NMR analysis (typically less than one month) and transferred to −80 °C for longer storage periods until subsequent NMR studies were performed. The samples were aliquoted in several containers before being frozen.

### 3.3. NMR Experiments

The NMR spectra were recorded on a Bruker Avance III 400 MHz spectrometer for the first quick diagnosis and on a Bruker Avance Neo 600 spectrometer for additional experiments. Both spectrometers were using 5 mm inverse detection multinuclear probes equipped with gradients on the z-axis. The samples were run in 5 mm Wilmad 507 NMR tubes. Before NMR analysis, the samples were allowed to reach room temperature (typically one hour) and centrifuged at 7000 rpm for 10 min. To 0.9 mL urine, 0.1 mL of a stock solution of 6 mM sodium 3-(trimethylsilyl)-[2,2,3,3-d_4_]-1-propionate (TSP) in KH_2_PO_4_/KOH/D_2_O buffer (Bruker Biospin) was added. The ^1^H-NMR spectra were recorded with NOSEY water presaturation. For initial diagnosis the NMR spectra have been recorded at 400 MHz in quantitative conditions with a pulse sequence using 32 scans, a 90° pulse, 30 s relaxation delay, 3 s CW irradiation, and 4 s acquisition time as previously described [14,15,16,17,20,76]. The second NMR analysis on another aliquot was performed at 600 MHz with pulse sequences and parameters as delivered with Bruker Biospin IVDr methods (V.2.0), which allow fast acquisition of ^1^H-NMR spectra using 32 scans, a 90° pulse, 4 s relaxation delay with simultaneous CW irradiation, and 2.7 s acquisition time with an ERETIC type of signal as the quantitation reference and J-Resolved NMR spectra for signal assignment support (4 min total experimental time for each type of spectrum) [77]. The IVDr raw NMR data have been processed with the commercial B.I.Quant-UR methods and models (Bruker Biospin). In addition to IVDr experiments, the second aliquot was also recorded at 600 MHz in fully relaxed conditions with identical quantitative ^1^H-NMR and COSY experiments (lasting 20 and 50 min respectively) as mentioned above for the 400 MHz instrument.

## 4. Conclusions

The paper presented metabolic monitoring by NMR of five cases of MMA patients. The compiled data from a large number of MMA samples presented in this paper should contribute to building up a better-defined model of targeted metabolic ranges useful for evaluating metabolic status. Moreover, the constant association of levels of MMAc and Gly with the type of mutation shows the potential of NMR to assign subtle differences within the same category of metabolic disorder and supports the idea of continuing to increase the targeted metabolic ranges for as many metabolites as possible.

The existence of completely asymptomatic cases of MMA and other metabolic disorders supports the idea that running at least one occasional NMR urine test for any individual regardless of age is a valuable health control check. Additionally, this advice may be even more important in some particular situations, such as just before or early during pregnancy.

## Figures and Tables

**Table 1:** Averaged absolute concentrations (mmol/L urine) and ranges for individual values (in brackets) for presently monitored MMA patients and previously reported controls. The number of samples n ^1^ in which a particular metabolite is detected may be lower than the total number of samples investigated.

Averaged Absolute Concentrations and Ranges for MMA Patients (mmol/L)	Concentrations and Ranges for Controls Previously Reported (mmol/L)
**Glycine (Gly)**	
1.916 (0.225–5.019) n = 9/9 Case 1 (7 do–9 mo) [MMUT] *1.101 (0.569–1.667) n = 3/3 Case 1—No Diet (7 do–4 mo)* *2.324 (0.225–5.019) n = 6/6 Case 1—Diet (19 do–9 mo)* 3.367 (0.771–7.253) n = 38/38 Case 2 (6 do–1½ yo) [MMUT] *2.741 (1.871–3.744) n = 4/4 Case 2—No Diet (11 do–1 mo)* *3.441 (0.771–7.253) n = 34/34 Case 2—Diet (3 mo–1½ yo)* 1.278 (0.175–3.411) n = 11/11 Case 3 (2½–3½ yo) [MMUT] 0.358 (0.152–0.835) n = 27/27 Case 4 (4½–5½ yo) [MMAA] *0.420 (0.152–0.835) n = 9/9 Case 4—No Diet (4y9mo–4y11mo)* *0.326 (0.202–0.506) n = 18/18 Case 4—Diet (5yo–5½ yo)* 0.194 (0.052–1.083) n = 95/95 Case 5 (2–11 yo) [MMAA] *0.116 (0.075–0.157) n = 5/5 Case 5—No Diet (2–11 yo)* *0.198 (0.052–1.083) n = 90/90 Case 5—Diet (2–11 yo)*	0.978 ± 0.790 (<28 do), 1.468 ± 1.185 (28–122 do) [57]; 1.581 ± 0.957 (1 do) [53].
**Methylmalonic Acid (MMAc)**	
22.349 (6.738–43.860) n = 9/9 Case 1 (7 do–9 mo) [MMUT] *27.317 (19.660–42.150) n = 3/3 Case 1—No Diet (7 do–4 mo)* *19.866 (6.738–43.860) n = 6/6 Case 1—Diet (19 do–9 mo)* 23.084 (8.872–48.500) n = 38/38 Case 2 (6 do–1½ yo) [MMUT] *16.608 (12.780–19.610) n = 4/4 Case 2—No Diet (11 do–1 mo)* *23.846 (8.872–48.500) n = 34/34 Case 2—Diet (3 mo–1½ yo)* 22.848 (6.542–36.580) n = 11/11 Case 3 (2½–3½ yo) [MMUT] 2.989 (0.174–18.960) n = 27/27 Case 4 (4½–5½ yo) [MMAA] *7.352 (1.607–18.960) n = 9/9 Case 4—No Diet (4y9mo–4y11mo)* *0.807 (0.174–3.437) n = 18/18 Case 4—Diet (5yo–5½ yo)* 4.085 (0.201–22.070) n = 95 Case 5 (2–11 yo) [MMAA] *15.253 (8.077–22.070) n = 5/5 Case 5—No Diet (2–11 yo)* *3.464 (0.201–9.079) n = 90/90 Case 5—Diet (2–11 yo)*	0.012 ± 0.017 (1 do) [53].
**Orotic Acid (Orot)**	
0.029 (0.007–0.066) n = 18/95 Case 5 (2–11 yo) [MMAA] *0.066 (0.066–0.066) n = 1/5 Case 5—No Diet (2–11 yo)* *0.027 (0.007–0.061) n = 17/90 Case 5—Diet (2–11 yo)*	
**Orotidine (Orotid)**	
0.082 (0.035–0.047) n = 19/95 Case 5 (2–11 yo) [MMAA] *0.097 (0.097–0.097) n = 1/5 Case 5—No Diet (2–11 yo)* *0.081 (0.035–0.132) n = 18/90 Case 5—Diet (2–11 yo)*	
**L-Carnitine (Car)**	
0.247 (0.147–0.333) n = 6/9 Case 1 (7 do–9 mo) [MMUT] *0.0 n = 0/3 Case 1—No Diet (7 do–4 mo)* *0.247 (0.147–0.333) n = 6/6 Case 1—Diet (19 do–9 mo)* 0.538 (0.156–1.334) n = 34/38 (6 do–1½ yo) [MMUT] *0.0 n = 0/4 Case 2—No Diet (11 do–1 mo)* *0.538 (0.156–1.334) n = 34/34 Case 2—Diet (3 mo–1½ yo)* 0.218 (0.096–0.456) n = 5/11 Case 3 (2½–3½ yo) [MMUT] 0.299 (0.068–0.782) n = 17/27 Case 4 (4½–5½ yo) [MMAA] *0.0 n = 0/9 Case 4—No Diet (4y9mo–4y11mo)* *0.299 (0.068–0.782) n = 17/18 Case 4—Diet (5yo–5½ yo)* 0.221 (0.024–0.667) n = 13/28 Case 5 (2–11 yo) [MMAA] *0.0 n = 0/5 Case 5—No Diet (2–11 yo)* *0.221 (0.024–0.667) n = 13/23 Case 5—Diet (2–11 yo)*	0.009 ± 0.007 (1 do) [53].
**Propionylcarnitine (PrCar)**	
0.715 (0.156–1.390) n = 6/9 Case 1 (7 do–9 mo) [MMUT] *0.0 n = 0/3 Case 1—No Diet (7 do–4 mo)* *0.715 (0.156–1.390) n = 6/6 Case 1—Diet (19 do–9 mo)* 0.003 (0.001–0.008) n = 34/38 Case 2 (6 do–1½ yo) [MMUT] *0.0 n = 0/4 Case 2—No Diet (11 do–1 mo)* *0.922 (0.253–2.266) n = 34/34 Case 2—Diet (3 mo–1½ yo)* 0.361 (0.182–0.612) n = 5/11 Case 3 (2½–3½ yo) [MMUT] 0.099 (0.036–0.245) n = 17/27 Case 4 (4½–5½ yo) [MMAA] *0.0 n = 0/9 Case 4—No Diet (4y9mo–4y11mo)* *0.099 (0.036–0.245) n = 17/18 Case 4—Diet (5yo–5½ yo)* 0.128 (0.044–0.199) n = 11/28 Case 5 (2–11 yo) [MMAA] *0.0 n = 0/5 Case 5—NoDiet (2–11 yo)* *0.128 (0.044–0.199) n = 11/23 Case 5—Diet (2–11 yo)*	<1.20 [8]; 0.002 (1 do) [53].

^1^ For a situation in which the number of reported samples is smaller than the total number of samples (n = Y/X with Y < X) it means that there were X-Y situations for which the metabolite was not detected (i.e., it was either zero or below the limit of detection).

**Table 2:** Averaged relative concentrations (mmol/mol Crn) and ranges for individual values (in brackets) for presently monitored MMA patients and previously reported controls. The number of samples n ^1^ in which a particular metabolite is detected may be lower than the total number of samples investigated.

Averaged Relative Concentrations and Ranges for MMA Patients (mmol/mol Crn)	Concentrations and Ranges for Controls Previously Reported (mmol/mol Crn)
**Glycine (Gly)**	
2322 (120–7300) n = 9/9 Case 1 (7 do–9 mo) [MMUT] *833 (250–1300) n = 3/3 Case 1—No Diet (7 do–4 mo)* *3067 (120–7300) n = 6/6 Case 1—Diet (19 do–9 mo)* 2277 (680–4400) n = 38/38 Case 2 (6 do–1½ yo) [MMUT] *3100 (2400–3900) n = 4/4 Case 2—No Diet (11 do–1 mo)* *2180 (680–4400) n = 34/34 Case 2—Diet (3 mo–1½ yo)* 602 (140–1400) n = 11/11 Case 3 (2½–3½ yo) [MMUT] 148 (69–330) n = 27/27 Case 4 (4½–5½ yo) [MMAA] *211 (110–330) n = 9/9 Case 4—No Diet (4y9mo–4y11mo)* *117 (69–240) n = 18/18 Case 4—Diet (5yo–5½ yo)* 66 (34–240) n = 79/95 Case 5 (2–11 yo) [MMAA] *41 (38–46) n = 3/5 Case 5—No Diet (2–11 yo)* *67 (34–240) n = 76/90 Case 5—Diet (2–11 yo)*	106 (44–300) (19–67 yo) [58]; 362.1 (102.6–853.6) (1 do) [53]; 283–1097 (0–1 mo), 210–743 (1–6 mo), 114–445 (6–12 mo), 110–356 (1–2 yo), 11–326 (2–4 yo), 91–246 (4–7 yo), 64–236 (7–13 yo), 43–173 (>13 yo) [8]; 113–1427 (0–1 mo), 147–570 (2–12 mo), 66–453 (1–8 yo), 51–238 (9–18 yo) [51].
**Methylmalonic Acid (MMAc)**	
16111 (7000–25000) n = 9/9 Case 1 (7 do–9 mo) [MMUT] *17000 (15000–18000) n = 3/3 Case 1—No Diet (7 do–4 mo)* *15667 (7000–25000) n = 6/6 Case 1—Diet (19 do–9 mo)* 15350 (5600–28000) n = 38/38 Case 2 (6 do–1½ yo) [MMUT] *20000 (14000–28000) n = 4/4 Case 2—No Diet (11 do–1 mo)* *14803 (5600–24000) n = 34/34 Case 2—Diet (3 mo–1½ yo)* 11645 (5600–25000) n = 11/11 Case 3 (2½–3½ yo) [MMUT] 1624 (76–14000) n = 27/27 Case 4 (4½–5½ yo) [MMAA] *4369 (760–14000) n = 9/9 Case 4—No Diet (4y9mo–4y11mo)* *251 (76–690) n = 18/18 Case 4—Diet (5yo–5½ yo)* 1259 (130–7900) n = 95/95 Case 5 (2–11 yo) [MMAA] *4980 (3200–7900) n = 5/5 Case 5—No Diet (2–11 yo)* *1052 (130–3800) n = 90/90 Case 5—Diet (2–11 yo)*	1–11 (0–4 mo), 2–13 (4–24 mo), 1–4 (2–10 yo), 0–4 (>10 yo) [8]; 0.58–3.56 [54]; 1.9 (0.7–3.5) (19–67 yo) [58]; 2.79 (0.42–19.7) (1 do) [53].
**Orotic Acid (Orot)**	
8 (5–16) n = 18/95 Case 5 (2–11 yo) [MMAA] *22 n = 1/5 Case 5—No Diet (2–11 yo)* *12 (2–26) n = 17/90 Case 5—Diet (2–11 yo*	1.85 (0–5) (21 do) [59].
**Orotidine (Orotid)**	
35 (21–55) n = 19/95 Case 5 (2–11 yo) [MMAA] *32 n = 1/5 Case 5—No Diet (2–11 yo)* *36 (21–55) n = 18/90 Case 5—Diet (2–11 yo)*	
**l-Carnitine (Car)**	
346 (189–838) n = 6/9 Case 1 (7 do–9 mo) [MMUT] *0 n = 0/3 Case 1—No Diet (7 do–4 mo)* *346 (189–838) n = 6/6 Case 1—Diet (19 do–9 mo)* 520 (162–1028) n = 34/38 Case 2 (6 do–1½ yo) [MMUT] *0 n = 0/4 Case 2—No Diet (11 do–1 mo)* *520 (162–1082) n = 34/34 Case 2—Diet (3 mo–1½ yo)* 153 (95–226) n = 5/11 Case 3 (2½–3½ yo) [MMUT] 165 (40–342) n = 17/27 Case 4 (4½–5½ yo) [MMAA] *0 n = 0/9 Case 4—No Diet (4y9mo–4y11mo)* *165 (40–342) n = 17/18 Case 4—Diet (5yo–5½ yo)* 109 (15–429) n = 13/28 Case 5 (2–11 yo) [MMAA] *0 n = 0/5 Case 5—No Diet (2–11 yo)* *109 (15–429) n = 13/23 Case 5—Diet (2–11 yo)*	5.0 (0.7–16.4) (19–67 yo) [58]; 2.01 (0.79–5.67) (1 do) [53].
**Propionylcarnitine (PrCar)**	
700 (206–1186) n = 6/9 Case 1 (7 do–9 mo) [MMUT] *0 n = 0/3 Case 1—No Diet (7 do–4 mo)* *700 (206–1186) n = 6/6 Case 1—Diet (19 do–9 mo)* 902 (404–1624) n = 34/38 Case 2 (6 do–1½ yo) [MMUT] *0 n = 0/4 Case 2—No Diet (11 do–1 mo)* *902 (404–1624) n = 34/34 Case 2—Diet (3 mo–1½ yo)* 262 (187–356) n = 5/11 Case 3 (2½–3½ yo) [MMUT] 55 (21–106) n = 17/27 Case 4 (4½–5½ yo) [MMAA] *0 n = 0/9 Case 4—No Diet (4y9mo–4y11mo)* *55 (21–106) n = 17/18 Case 4—Diet (5yo–5½ yo)* 65 (40–124) n = 11/28 Case 5 (2–11 yo) [MMAA] *0 n = 0/5 Case 5—No Diet (2–11 yo)* *65 (40–124) n = 11/23 Case 5—Diet (2–11 yo)*	0.07 (0.01–0.20) (19–67 yo) [58]; 0.03 (0.01–0.07) (1 do) [53].

^1^ For a situation in which the number of reported samples is smaller than the total number of samples (n = Y/X with Y < X) it means that there were X-Y situations for which the metabolite was not detected (i.e., it was either zero or below the limit of detection).

## Supplementary Materials

The following are available online, Figure S1: ^1^H-NMR spectrum at 600 MHz in fast acquisition mode and ERETIC signal (at 12.0 ppm) for a urine sample from a 5 ½ y.o. boy with MMA (Case 5), Figure S2: ^1^H-NMR spectrum at 600 MHz in fully relaxed acquisition mode for a urine sample from a 5 ½ y.o. boy with MMA (Case 5), Figure S3: ^1^H J-Resolved NMR spectrum at 600 MHz for a urine sample from a 5 ½ y.o. boy with MMA (Case 5), used as support for assignment of signals in the ^1^H-NMR spectra, Figure S4: H-H COSY NMR spectrum at 600 MHz for a urine sample from a 5 ½ y.o. boy with MMA (Case 5), used as support for assignment of signals in the ^1^H-NMR spectra, Table S1: Averaged absolute concentrations (mmol/L urine) and ranges for individual values (in brackets) for presently monitored MMA patients and previously reported controls. The number of samples n^1^ in which a particular metabolite is detected may be lower than the total number of samples investigated, Table S2: Averaged relative concentrations (mmol/mol Crn) and ranges for individual values (in brackets) for presently monitored MMA patients and previously reported controls. The number of samples n^1^ in which a particular metabolite is detected may be lower than the total number of samples investigated.
