# Supplementary material for: Monitoring Methylmalonic Aciduria by NMR Urinomics
Source: Molecules. 2020 Nov 14;25(22):5312. doi: 10.3390/molecules25225312 (PMC7697698; doi:10.3390/molecules25225312)
Supplement: Supplementary file 1 [file molecules-25-05312-s001.pdf]

Supplementary material for:

Monitoring Methylmalonic Aciduria by NMR Urinomics

Alina Nicolescu [1,2,*], Daniela Blanita [3], Chiril Boiciuc [3], Victoria Hlistun [3], Mihaela Cristea [1], Dorina Rotaru [4], Ludmila Pinzari [3], Ana Oglinda [5], Adela Stamati [5], Isabela Tarcomnicu [6], Andreea Tutulan-Cunita [6], Danae Stambouli [6], Sergiu Gladun [3], Ninel Revenco [5], Natalia Uşurelu [3,*] and Calin Deleanu [1,2,*]

[1] "Petru Poni" Institute of Macromolecular Chemistry, Romanian Academy, Aleea Grigore Ghica Voda 41A, RO-700487 Iasi, Romania; ciobanu.mihaela20@yahoo.com

[2] "C. D. Nenitescu" Centre of Organic Chemistry, Romanian Academy, Spl. Independentei 202B, RO-060023 Bucharest, Romania

[3] Institute of Mother and Child, Str. Burebista 93, MD-2062 Chisinau, Republic of Moldova; blanita.daniela@gmail.com (D.B.); tervenom@gmail.com (C.B.); hlistunvica@yahoo.com (V.H.); liudmilapinzari@rambler.ru (L.P.); gladunsergiu@gmail.com (S.G.)

[4] "Gheorghe Palade" City Clinical Hospital, *Str. Melestiu 20, MD-2001* Chisinau, Republic of Moldova; dorinarotaru@rambler.ru

[5] "Nicolae Testemitanu" State University of Medicine and Pharmacy, Bd. Stefan cel Mare si Sfint 165, MD-2004 Chisinau, Republic of Moldova; ana.oglinda@usmf.md (A.O.); adela.stamati@usmf.md (A.S.); ninel.revenco@usmf.md (N.R.)

[6] Cytogenomic Medical Laboratory, Calea Floreasca 35, RO-014453 Bucharest, Romania; isa.tarcomnicu@gmail.com (I.T.); cunita@cytogenomic.ro (A.T.-C.); stambouli@cytogenomic.ro (D.S.)

* Correspondence: alina@icmpp.ro (A.N.); natalia.usurelu@mama-copilul.md (N.U.); calind@ccocdn.ro or calin.deleanu@yahoo.com (C.D.); Tel.: +40-744-340-456 (C.D.)

The full set of NMR experiments is exemplified below (figures S1-S4) for a urine sample from a 5 ½ y.o. boy with MMA (Case 5).

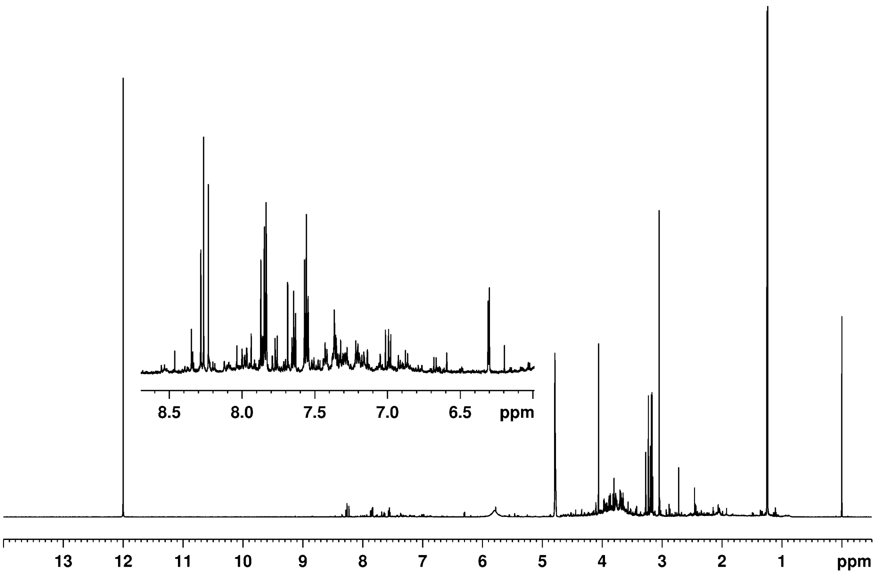

Figure S1. ^{1}H-NMR spectrum at 600 MHz in fast acquisition mode and ERETIC signal (at 12.0 ppm) for a urine sample from a 5 ½ y.o. boy with MMA (Case 5).

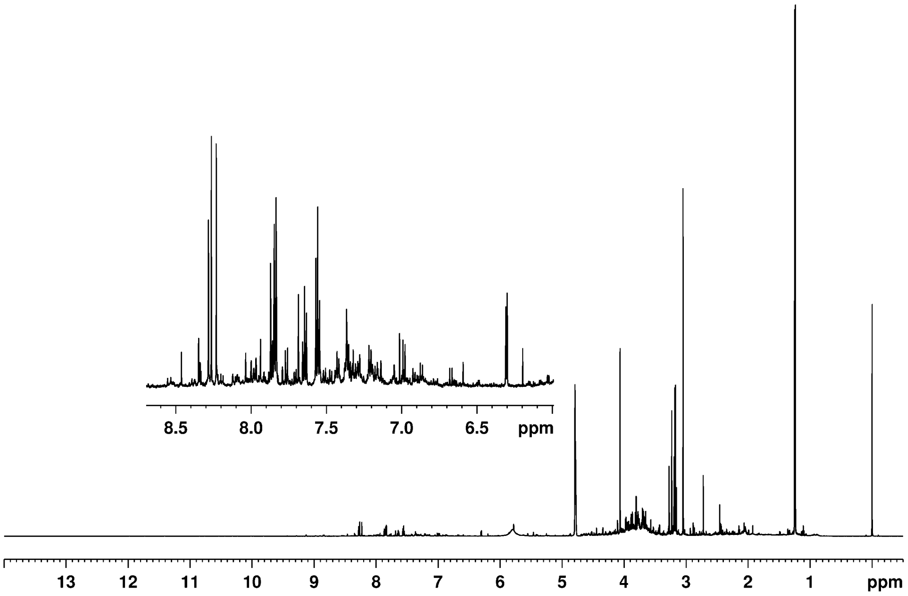

Figure S2. ^{1}H-NMR spectrum at 600 MHz in fully relaxed acquisition mode for a urine sample from a 5 ½ y.o. boy with MMA (Case 5).

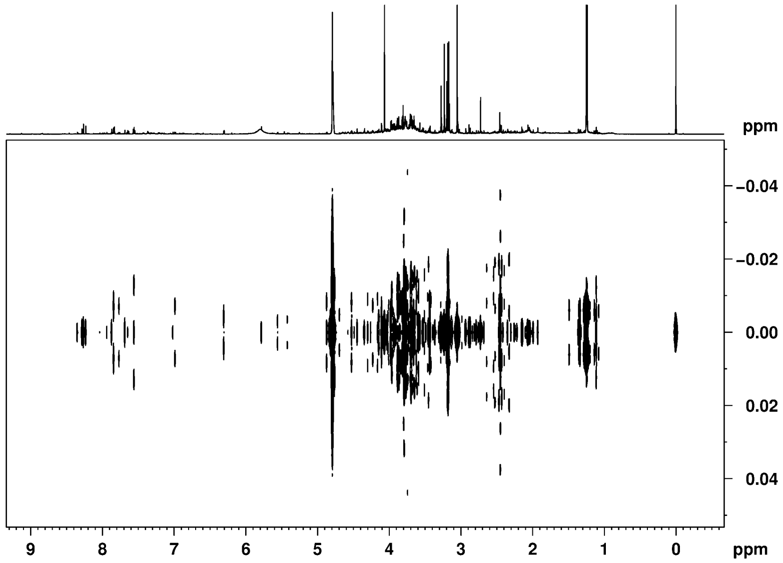

Figure S3. ^{1}H J-Resolved NMR spectrum at 600 MHz for a urine sample from a 5 ½ y.o. boy with MMA (Case 5), used as support for assignment of signals in the ^{1}H-NMR spectra.

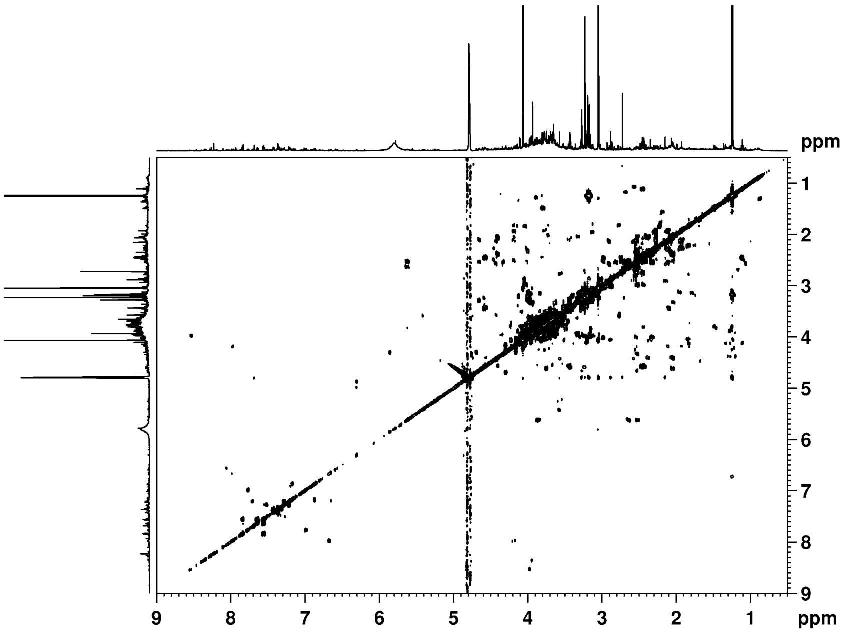

Figure S4. H-H COSY NMR spectrum at 600 MHz for a urine sample from a 5 ½ y.o. boy with MMA (Case 5), used as support for assignment of signals in the ^{1}H-NMR spectra.

The full sets of concentrations for both MMA specific and non-specific metabolites as determined by NMR urinomics and previously reported reference ranges are presented below in tables S1 and S2.

Table S1. Averaged absolute concentrations (mmol/L urine) and ranges for individual values (in brackets) for presently monitored MMA patients and previously reported controls. The number of samples n[1] in which a particular metabolite is detected may be lower than the total number of samples investigated.

**Averaged Absolute Concentrations and Ranges for MMA Patients (mmol/L)**	**Concentrations and Ranges for Controls Previously Reported (mmol/L)**
**Creatinine (Crn)**	
1.348 (0.515-2.295) n=9/9 Case 1 (7 do-9 mo) [MMUT]<br>*1.574 (1.120-2.295) n=3/3 Case 1 – No Diet (7 do-4 mo)*<br>*1.235 (0.515-1.954) n=6/6 Case 1 – Diet (19 do-9 mo)*<br>1.612 (0.577-3.638) n=38/38 Case 2 (6 do-1½ yo) [MMUT]<br>*0.892 (0.583-1.310) n=4/4 Case 2 – No Diet (11 do-1 mo)*<br>*1.697 (0.577-3.638) n=34/34 Case 2 – Diet (3 mo-1½ yo)*<br>2.383 (0.364-4.409) n=11/11 Case 3 (2½-3½ yo) [MMUT]<br>2.677 (1.190-4.964) n=27/27 Case 4 (4½-5½ yo) [MMAA]<br>*1.997 (1.190-3.101) n=9/9 Case 4 – No Diet (4y9mo-4y11mo)*<br>*3.018 (1.791-4.964) n=18/18 Case 4 – Diet (5-5½ yo)*<br>3.518 (1.039-9.950) n=95/95 Case 5 (2-11 yo) [MMAA]<br>*3.176 (1.990-5.007) n=5/5 Case 5– No Diet (2-11 yo)*<br>*3.537 (1.039-9.950) n=90/90 Case 5 – Diet (2-11 yo)*	2.3 (0.980-10.520) (0-30 do), M/F 10.460 ± 2.040 / 6.770 ± 0.460 (>18 yo) [47];<br>10.75 (2.42-33.61) (24-59) yo [15];<br>15.51 (2.16-42.03) (25-67) yo [14];<br>1.081±1.423 (<28 do), 1.622±2.135 (28-122 do) [57];<br>14.743±9.797 (19-67 yo) [58];<br>0.37 (0.09-1.46) (21 do) [59];<br>4.952 (1.083-16.760) (1 do) [53].

**Valine (Val)**	
0.047 (0.011-0.115) n=9/9 Case 1 (7 do-9 mo) [MMUT]<br>*0.053 (0.017-0.115) n=3/3 Case 1 – No Diet (7 do-4 mo)*<br>*0.044 (0.011-0.108) n=6/6 Case 1 – Diet (19 do-9 mo)*<br>0.025 (0.001-0.182) n=37/38 Case 2 (6 do-1½ yo) [MMUT]<br>*0.071 (0.024-0.182) n=4/4 Case 2 – No Diet (11 do-1 mo)*<br>*0.019 (0.001-0.045) n=33/34 Case 2 – Diet (3 mo-1½ yo)*<br>0.011 (0.001-0.023) n=11/11 Case 3 (2½ - 3½ yo) [MMUT]<br>0.008 (0.001-0.021) n=27/27 Case 4 (4½-5½ yo) [MMAA]<br>*0.011 (0.002-0.019) n=9/9 Case 4 – No Diet (4y9mo- 4y11mo)*<br>*0.006 (0.001-0.011) n=18/18 Case 4 – Diet (5yo-5½ yo)*<br>0.009 (0.002-0.020) n=88/95 Case 5 (2-11 yo) [MMAA]<br>*0.010 (0.004-0.016) n=2/5 Case 5 – No Diet (2-11 yo)*<br>*0.009 (0.002-0.020) n=86/90 Case 5 – Diet (2-11 yo)*	0.16 (0.08-0.28) (24-59 yo) [15];<br>0.12 (0.02-0.21) (25-67 yo) [14];<br>0.031±0.026 (1 do) [53].
**Lactic acid (Lac)**	
0.815 (0.126-3.368) n=9/9 Case 1 (7 do-9 mo) [MMUT]<br>*0.398 (0.260-0.482) n=3/3 Case 1 – No Diet (7 do-4 mo)*<br>*1.023 (0.126-3.368) n=6/6 Case 1 – Diet (19 do-9 mo)*<br>0.283 (0.084-2.939) n=38/38 Case 2 (6 do-1½ yo) [MMUT]<br>*1.207 (0.189-2.939) n=4/4 Case 2 – No Diet (11 do-1 mo)*<br>*0.175 (0.084-0.582) n=34/34 Case 2 – Diet (3 mo-1½ yo)*<br>0.152 (0.050-0.365) n=11/11 Case 3 (2½ - 3½ yo) [MMUT]<br>0.079 (0.024-0.242) n=27/27 Case 4 (4½-5½ yo) [MMAA]<br>*0.100 (0.025-0.242) n=9/9 Case 4 – No Diet (4y9mo- 4y11mo)*<br>*0.068 (0.024-0.161) n=18/18 Case 4– Diet (5yo-5½ yo)*<br>0.086 (0.033-0.266) n=95/95 Case 5 (2-11 yo) [MMAA]<br>*0.110 (0.061-0.170) n=5/5 Case 5– No Diet (2-11 yo)*<br>*0.085 (0.033-0.266) n=90/90 Case 5– Diet (2-11 yo)*	0.57 (0.16-1.38) (24-59 yo) [15];<br>0.50 (0.09-1.35) (25-67 yo) [14];<br>0.742±0.780 (1 do) [53].
**Alanine (Ala)**	
0.235 (0.107-0.324) n=9/9 Case 1 (7 do-9 mo) [MMUT]<br>*0.230 (0.163-0.299) n=3/3 Case 1 – No Diet (7 do-4 mo)*<br>*0.237 (0.107-0.324) n=6/6 Case 1 – Diet (19 do-9 mo)*<br>0.242 (0.082-0.692) n=38/38 Case 2 (6 do-1½ yo) [MMUT]<br>*0.350 (0.257-0.413) n=4/4 Case 2 – No Diet (11 do-1 mo)*<br>*0.230 (0.082-0.692) n=34/34 Case 2 – Diet (3 mo-1½ yo)*<br>0.167 (0.031-0.434) n=11/11 Case 3 (2½-3½ yo) [MMUT]<br>0.135 (0.023-0.238) n=27/27 Case 4 (4½-5½ yo) [MMAA]<br>*0.125 (0.023-0.238) n=9/9 Case 4 – No Diet (4y9mo- 4y11mo)*<br>*0.140 (0.094-0.234) n=18/18 Case 4 – Diet (5yo-5½ yo)*<br>0.090 (0.090-0.019) n=95/95 Case 5 (2-11 yo) [MMAA]<br>*0.045 (0.027-0.073) n=5/5 Case 5 – No Diet (2-11 yo)*<br>*0.092 (0.019-0.164) n=90/90 Case 5 – Diet (2-11 yo)*	0.54 (0.11-1.58) [15];<br>0.63 (0.09-1.28) (25-67 yo) [14];<br>0.354±0.261 (1 do) [53];<br>0.157±0.147 (<28 do), 0.236±0.220 (28-122 do) [57].
**Pyruvic acid (Pyr)**	
0.225 (0.001-0.890) n=9/9 Case 1 (7 do-9 mo) [MMUT]<br>*0.154 (0.042-0.274) n=3/3 Case 1 – No Diet (7 do-4 mo)*<br>*0.260 (0.001-0.890) n=6/6 Case 1 – Diet (19 do-9 mo)*<br>0.063 (0.015-0.373) n=38/38 Case 2 (6 do-1½ yo) [MMUT]<br>*0.181 (0.056-0.373) n=4/4 Case 2 – No Diet (11 do-1 mo)*<br>*0.049 (0.015-0.137) n=34/34 Case 2 – Diet (3 mo-1½ yo)*<br>0.028 (0.013-0.046) n=11/11 Case 3 (2½-3½ yo) [MMUT]<br>0.038 (0.015-0.196) n=27/27 Case 4 (4½-5½ yo) [MMAA]	0.48 (0.07-1.44) (24-59 yo) [15];<br>0.62 (0.08-1.99) (25-67 yo) [14];<br>0.164±0.096 (1 do) [53].

0.051 (0.015-0.196) n=9/9 Case 4 – No Diet (4y9mo- 4y11mo)	
0.032 (0.021-0.058) n=18/18 Case 4 – Diet (5yo-5½ yo)	
0.033 (0.013-0.060) n=95/95 Case 5 (2-11 yo) [MMAA]	
0.022 (0.019-0.027) n=5/5 Case 5 – No Diet (2-11 yo)	
0.033 (0.013-0.060) n=90/90 Case 5 – Diet (2-11 yo)	
**Citric acid (Cit)**	
0.430 (0.073-1.068) n=9/9 Case 1 (7 do-9 mo) [MMUT]	
0.412 (0.133-0.822) n=3/3 Case 1 – No Diet (7 do-4 mo)	
0.440 (0.073-1.068) n=6/6 Case 1 – Diet (19 do-9 mo)	
0.790 (0.103-2.418) n=38/38 Case 2 (6 do-1½ yo) [MMUT]	
0.345 (0.103-0.565) n=4/4 Case 2 – No Diet (11 do-1 mo)	
0.843 (0.113-2.418) n=34/34 Case 2 – Diet (3 mo-1½ yo)	2.36 (0.51-7.71) (24-59 yo) [15];
0.973 (0.088-3.706) n=11/11 Case 3 (2½-3½ yo) [MMUT]	2.95 (0.68-5.87) (25-67 yo) [14];
0.671 (0.035-1.619) n=25/27 Case 4 (4½-5½ yo) [MMAA]	1.833±1.666 (1 do) [53].
0.314 (0.035-0.702) n=7/9 Case 4 – No Diet (4y9mo- 4y11mo)	
0.850 (0.480-1.619) n=18/18 Case 4 – Diet (5yo-5½ yo)	
0.111 (0.010-0.666) n=95/95 Case 5 (2-11 yo) [MMAA]	
0.030 (0.016-0.051) n=5/5 Case 5– No Diet (2-11 yo)	
0.116 (0.010-0.666) n=90/90 Case 5 – Diet (2-11 yo)	
**Dimethylamine (DMA)**	
0.122 (0.057-0.207) n=9/9 Case 1 (7 do-9 mo) [MMUT]	
0.101 (0.057-0.136) n=3/3 Case 1 – No Diet (7 do-4 mo)	
0.133 (0.067-0.207) n=6/6 Case 1 – Diet (19 do-9 mo)	
0.173 (0.055-0.382) n=38/38 Case 2 (6 do-1½ yo) [MMUT]	
0.104 (0.055-0.158) n=4/4 Case 2 – No Diet (11 do-1 mo)	0.34 (0.05-0.95) (24-59 yo) [15];
0.181 (0.066-0.382) n=34/34 Case 2 – Diet (3 mo-1½ yo)	0.41 (0.06-1.40) (25-67 yo) [14];
0.224 (0.035-0.415) n=11/11 Case 3 (2½-3½ yo) [MMUT]	0.226±0.248 (<28 do), 0.339±0.372
0.213 (0.068-0.390) n=27/27 Case 4 (4½-5½ yo) [MMAA]	(28-122 do) [57].
0.148 (0.068-0.258) n=9/9 Case 4 – No Diet (4y9mo- 4y11mo)	
0.246 (0.133-0.390) n=18/18 Case 4 – Diet (5yo-5½ yo)	
0.209 (0.078-0.428) n=95/95 Case 5 (2-11 yo) [MMAA]	
0.131 (0.102-0.168) n=5/5 Case 5 – No Diet (2-11 yo)	
0.214 (0.078-0.428) n=90/90 Case 5 – Diet (2-11 yo)	
**Trimethylamine (TMA)**	
0.006 (0.002-0.014) n=9/9 Case 1 (7 do-9 mo) [MMUT]	
0.008 (0.004-0.014) n=3/3 Case 1 – No Diet (7 do-4 mo)	
0.004 (0.002-0.008) n=6/6 Case 1 – Diet (19 do-9 mo)	
0.021 (0.001-0.480) n=38/38 Case 2 (6 do-1½ yo) [MMUT]	
0.004 (0.002-0.006) n=4/4 Case 2 – No Diet (11 do-1 mo)	
0.023 (0.001-0.480) n=34/34 Case 2 – Diet (3 mo-1½ yo)	
0.006 (0.001-0.024) n=11/11 Case 3 (2½-3½ yo) [MMUT]	
0.006 (0.001-0.035) n=27/27 Case 4 (4½-5½ yo) [MMAA]	
0.003 (0.001-0.005) n=9/9 Case 4 – No Diet (4y9mo- 4y11mo)	
0.007 (0.003-0.035) n=18/18 Case 4 – Diet (5yo-5½ yo)	
0.008 (0.001-0.150) n=95/95 Case 5 (2-11 yo) [MMAA]	
0.002 (0.001-0.003) n=5/5 Case 5 – NoDiet (2-11 yo)	
0.009 (0.001-0.150) n=90/90 Case 5 – Diet (2-11 yo)	
**Glycine (Gly)**	
1.916 (0.225-5.019) n=9/9 Case 1 (7 do-9 mo) [MMUT]	0.978±0.790 (<28 do), 1.468±1.185
1.101 (0.569-1.667) n=3/3 Case 1 – No Diet (7 do-4 mo)	(28-122 do) [57];
2.324 (0.225-5.019) n=6/6 Case 1 – Diet (19 do-9 mo)	1.581±0.957 (1 do) [53].

3.367 (0.771-7.253) n=38/38 Case 2 (6 do-1½ yo) [MMUT]	
2.741 (1.871-3.744) n=4/4 Case 2 – No Diet (11 do-1 mo)	
3.441 (0.771-7.253) n=34/34 Case 2 – Diet (3 mo-1½ yo)	
1.278 (0.175-3.411) n=11/11 Case 3 (2½-3½ yo) [MMUT]	
0.358 (0.152-0.835) n=27/27 Case 4 (4½-5½ yo) [MMAA]	
0.420 (0.152-0.835) n=9/9 Case 4 – No Diet (4y9mo- 4y11mo)	
0.326 (0.202-0.506) n=18/18 Case 4 – Diet (5yo-5½ yo)	
0.194 (0.052-1.083) n=95/95 Case 5 (2-11 yo) [MMAA]	
0.116 (0.075-0.157) n=5/5 Case 5 – No Diet (2-11 yo)	
0.198 (0.052-1.083) n=90/90 Case 5 – Diet (2-11 yo)	
**Hippuric acid (Hip)**	
0.279 (0.030-1.156) n=7/9 Case 1 (7 do-9 mo) [MMUT]	
0.181 (0.082-0.327) n=3/3 Case 1 – No Diet (7 do-4 mo)	
0.352 (0.030-1.156) n=4/6 Case 1 – Diet (19 do-9 mo)	
0.235 (0.010-2.036) n=28/38 Case 2 (6 do-1½ yo) [MMUT]	
0.108 (0.062-0.158) n=3/4 Case 2 – No Diet (11 do-1 mo)	
0.250 (0.010-2.036) n=25/34 Case 2 – Diet (3 mo-1½ yo)	1.97 (0.38-5.84) (24-59 yo) [15];
0.292 (0.019-1.192) n=11/11 Case 3 (2½-3½ yo) [MMUT]	3.78 (0.53-27.80) (25-67 yo) [14];
1.036 (0.154-4.113) n=27/27 Case 4 (4½-5½ yo) [MMAA]	0.262±0.286 (1 do) [53].
0.706 (0.154-1.509) n=9/9 Case 4 – No Diet (4y9mo- 4y11mo)	
1.201 (0.164-4.113) n=18/18Case 4 – Diet (5yo-5½ yo)	
0.763 (0.056-5.027) n=95/95 Case 5 (2-11 yo) [MMAA]	
0.460 (0.287-0.687) n=5/5 Case 5 – No Diet (2-11 yo)	
0.780 (0.056-5.027) n=90/90 Case 5– Diet (2-11 yo)	
**Formic acid (For)**	
0.074 (0.031-0.156) n=9/9 Case 1 (7 do-9 mo) [MMUT]	
0.118 (0.067-0.156) n=3/3 Case 1 – No Diet (7 do-4 mo)	
0.052 (0.031-0.101) n=6/6 Case 1 – Diet (19 do-9 mo)	
0.062 (0.013-0.0234) n=38/38 Case 2 (6 do-1½ yo) [MMUT]	
0.130 (0.066-0.234) n=4/4 Case 2 – No Diet (11 do-1 mo)	
0.054 (0.013-0.149) n=34/34 Case 2 – Diet (3 mo-1½ yo)	
0.029 (0.007-0.069) n=11/11 Case 3 (2½-3½ yo) [MMUT]	0.43 (0.19-0.92) (24-59 yo) [15];
0.039 (0.007-0.072) n=27/27 Case 4 (4½-5½ yo) [MMAA]	0.38 (0.09-1.11) (25-67 yo) [14].
0.019 (0.007-0.039) n=9/9 Case 4 – No Diet (4y9mo- 4y11mo)	
0.049 (0.015-0.072) n=18/18 Case 4 – Diet (5yo-5½ yo)	
0.040 (0.007-0.103) n=95/95 Case 5 (2-11 yo) [MMAA]	
0.028 (0.022-0.040) n=5/5 Case 5 – No Diet (2-11 yo)	
0.041 (0.007-0.103) n=90/90 Case 5 – Diet (2-11 yo)	
**1-Methylguanidine**	
0.180 (0.071-0.401) n=9/9 Case 1 (7 do-9 mo) [MMUT]	
0.252 (0.152-0.401) n=3/3 Case 1 – No Diet (7 do-4 mo)	
0.144 (0.071-0.182) n=6/6 Case 1 – Diet (19 do-9 mo)	
0.181 (0.078-0.334) n=38/38 Case 2 (6 do-1½ yo) [MMUT]	
0.210 (0.136-0.334) n=4/4 Case 2 – No Diet (11 do-1 mo)	
0.178 (0.078-0.302) n=34/34 Case 2 – Diet (3 mo-1½ yo)	
0.217 (0.076-0.398) n=11/11 Case 3 (2½-3½ yo) [MMUT]	
0.190 (0.085-0.420) n=27/27 Case 4 (4½-5½ yo) [MMAA]	
0.159 (0.085-0.219) n=9/9 Case 4 – No Diet (4y9mo- 4y11mo)	
0.205 (0.132-0.420) n=18/18 Case 4– Diet (5yo-5½ yo)	
0.179 (0.069-0.326) n=95/95 Case 5 (2-11 yo) [MMAA]	
0.112 (0.080-0.158) n=5/5 Case 5 – No Diet (2-11 yo)	

0.183 (0.069-0.326) n=90/90 Case 5 – Diet (2-11 yo)	
**Betaine**	
0.917 (0.011-1.666) n=8/9 Case 1 (7 do-9 mo) [MMUT]	
1.110 (0.821-1.567) n=3/3 Case 1 – No Diet (7 do-4 mo)	
0.801 (0.011-1.666) n=5/6 Case 1 – Diet (19 do-9 mo)	
0.264 (0.050-1.293) n=38/38 Case 2 (6 do-1½ yo) [MMUT]	
0.652 (0.063-1.293) n=4/4 Case 2 – No Diet (11 do-1 mo)	
0.218 (0.050-0.849) n=34/34 Case 2 – Diet (3 mo-1½ yo)	0.619±0.585 (<28 do), 0.929±0.878
0.213 (0.079-0.527) n=11/11 Case 3 (2½-3½ yo) [MMUT]	(28-122 do) [57];
0.086 (0.011-0.226) n=27/27 Case 4 (4½-5½ yo) [MMAA]	0.781±0.312 (1 do) [53].
0.045 (0.013-0.065) n=9/9 Case 4 – No Diet (4y9mo- 4y11mo)	
0.106 (0.011-0.226) n=18/18 Case 4– Diet (5yo-5½ yo)	
0.129 (0.030-0.514) n=95/95 Case 5 (2-11 yo) [MMAA]	
0.057 (0.038-0.082) n=5/5 Case 5 – No Diet (2-11 yo)	
0.133 (0.030-0.514) n=90/90 Case 5– Diet (2-11 yo)	
**Creatine (Crea)**	
0.469 (0.047-1.530) n=9/9 Case 1 (7 do-9 mo) [MMUT]	
0.257 (0.135-0.480) n=3/3 Case 1 – No Diet (7 do-4 mo)	
0.574 (0.047-1.530) n=6/6 Case 1 – Diet (19 do-9 mo)	
1.563 (0.555-3.887) n=38/38 Case 2 (6 do-1½ yo) [MMUT]	
1.250 (0.555-2.765) n=4/4 Case 2 – No Diet (11 do-1 mo)	
1.600 (0.596-3.887) n=34/34 Case 2 – Diet (3 mo-1½ yo)	
0.685 (0.025-2.034) n=11/11 Case 3 (2½-3½ yo) [MMUT]	0.209±0.325 (1 do) [53].
0.208 (0.028-1.665) n=27/27 Case 4 (4½-5½ yo) [MMAA]	
0.312 (0.030-0.706) n=9/9 Case 4 – No Diet (4y9mo- 4y11mo)	
0.156 (0.028-1.665) n=18/18 Case 4 – Diet (5yo-5½ yo)	
0.367 (0.008-1.862) n=95/95 Case 5 (2-11 yo) [MMAA]	
0.118 (0.073-0.186) n=5/5 Case 5 – NoDiet (2-11 yo)	
0.381 (0.008-1.862) n=90/90 Case 5 – Diet (2-11 yo)	
***N,N*-Dimethylglycine**	
0.086 (0.042-0.167) n=9/9 Case 1 (7 do-9 mo) [MMUT]	
0.100 (0.061-0.164) n=3/3 Case 1 – No Diet (7 do-4 mo)	
0.080 (0.042-0.167) n=6/6 Case 1 – Diet (19 do-9 mo)	
0.034 (0.008-0.137) n=38/38 Case 2 (6 do-1½ yo) [MMUT]	
0.073 (0.040-0.137) n=4/4 Case 2 – No Diet (11 do-1 mo)	
0.029 (0.008-0.084) n=34/34 Case 2 – Diet (3 mo-1½ yo)	
0.036 (0.009-0.054) n=11/11 Case 3 (2½-3½ yo) [MMUT]	
0.052 (0.007-0.108) n=27/27 Case 4 (4½-5½ yo) [MMAA]	
0.029 (0.007-0.061) n=9/9 Case 4 – No Diet (4y9mo- 4y11mo)	
0.063 (0.038-0.108) n=18/18 Case 4 – Diet (5yo-5½ yo)	
0.044 (0.013-0.093) n=95/95 Case 5 (2-11 yo) [MMAA]	
0.027 (0.017-0.044) n=5/5 Case 5 – No Diet (2-11 yo)	
0.045 (0.013-0.093) n=90/90 Case 5 – Diet (2-11 yo)	
**Taurine**	
1.468 (0.166-5.929) n=9/9 Case 1 (7 do-9 mo) [MMUT]	
0.475 (0.166-1.054) n=3/3 Case 1 – No Diet (7 do-4 mo)	
1.965 (0.577-5.929) n=6/6 Case 1 – Diet (19 do-9 mo)	
1.022 (0.108-10.810) n=29/38 Case 2 (6 do-1½ yo) [MMUT]	5.526±5.919 (1 do) [53].
0.794 (0.114-1.371) n=3/4 Case 2 – No Diet (11 do-1 mo)	
1.048 (0.108-10.810) n=26/34 Case 2 – Diet (3 mo-1½ yo)	
2.010 (0.114-7.231) n=11/11 Case 3 (2½-3½ yo) [MMUT]	

0.313 (0.011-0.803) n=27/27 Case 4 (4½-5½ yo) [MMAA]	
0.045 (0.011-0.103) n=9/9 Case 4 – No Diet (4y9mo- 4y11mo)	
0.447 (0.069-0.803) n=18/18 Case 4 – Diet (5yo-5½ yo)	
1.128 (0.005-8.915) n=87/95 Case 5 (2-11 yo) [MMAA]	
0.126 (0.031-0.289) n=5/5 Case 5 – No Diet (2-11 yo)	
1.189 (0.005-8.915) n=82/90 Case 5 – Diet (2-11 yo)	
**Acetic acid**	
0.064 (0.032-0.101) n=9/9 Case 1 (7 do-9 mo) [MMUT]	
0.069 (0.053-0.085) n=3/3 Case 1 – No Diet (7 do-4 mo)	
0.061 (0.032-0.101) n=6/6 Case 1 – Diet (19 do-9 mo)	
0.079 (0.032-0.259) n=38/38 Case 2 (6 do-1½ yo) [MMUT]	
0.124 (0.054-0.259) n=4/4 Case 2 – No Diet (11 do-1 mo)	
0.074 (0.032-0.187) n=34/34 Case 2 – Diet (3 mo-1½ yo)	
0.050 (0.023-0.106) n=11/11 Case 3 (2½-3½ yo) [MMUT]	0.606±1.275 (<28do); 0.909±1.913 (28-122 do) [57].
0.037 (0.008-0.078) n=27/27 Case 4 (4½-5½ yo) [MMAA]	
0.027 (0.008-0.054) n=9/9 Case 4 – No Diet (4y9mo- 4y11mo)	
0.042 (0.023-0.078) n=18/18 Case 4 – Diet (5yo-5½ yo)	
0.061 (0.019-0.197) n=95/95 Case 5 (2-11 yo) [MMAA]	
0.042 (0.033-0.050) n=5/5 Case 5 – No Diet (2-11 yo)	
0.062 (0.019-0.197) n=90/90 Case 5 – Diet (2-11 yo)	
**Fumaric acid**	
0.024 (0.010-0.037) n=9/9 Case 1 (7 do-9 mo) [MMUT]	
0.019 (0.010-0.027) n=3/3 Case 1 – No Diet (7 do-4 mo)	
0.027 (0.015-0.037) n=6/6 Case 1 – Diet (19 do-9 mo)	
0.019 (0.005-0.097) n=38/38 Case 2 (6 do-1½ yo) [MMUT]	
0.050 (0.014-0.097) n=4/4 Case 2 – No Diet (11 do-1 mo)	
0.015 (0.005-0.034) n=34/34 Case 2 – Diet (3 mo-1½ yo)	
0.020 (0.003-0.061) n=11/11 Case 3 (2½-3½ yo) [MMUT]	0.060±0.042 (1 do) [53].
0.009 (0.002-0.050) n=27/27 Case 4 (4½-5½ yo) [MMAA]	
0.015 (0.008-0.050) n=9/9 Case 4 – No Diet (4y9mo- 4y11mo)	
0.006 (0.002-0.010) n=18/18 Case 4 – Diet (5yo-5½ yo)	
0.003 (0.001-0.016) n=95/95 Case 5 (2-11 yo) [MMAA]	
0.002 (0.002-0.003) n=5/5 Case 5 – No Diet (2-11 yo)	
0.003 (0.001-0.016) n=90/90 Case 5 – Diet (2-11 yo)	
**Succinic acid**	
0.118 (0.009-0.360) n=9/9 Case 1 (7 do-9 mo) [MMUT]	
0.117 (0.075-0.200) n=3/3 Case 1 – No Diet (7 do-4 mo)	
0.118 (0.009-0.360) n=6/6 Case 1 – Diet (19 do-9 mo)	
0.106 (0.037-0.296) n=38/38 Case 2 (6 do-1½ yo) [MMUT]	
0.112 (0.048-0.167) n=4/4 Case 2 – No Diet (11 do-1 mo)	
0.106 (0.037-0.296) n=34/34 Case 2 – Diet (3 mo-1½ yo)	
0.104 (0.012-0.277) n=11/11 Case 3 (2½-3½ yo) [MMUT]	0.094±0.092 (<28 do), 0.140±0.138 (28-122 do) [57].
0.131 (0.021-0.349) n=27/27 Case 4 (4½-5½ yo) [MMAA]	
0.048 (0.021-0.085) n=9/9 Case 4 – No Diet (4y9mo- 4y11mo)	
0.172 (0.109-0.349) n=18/18 Case 4 – Diet (5yo-5½ yo)	
0.048 (0.009-0.157) n=95/95 Case 5 (2-11 yo) [MMAA]	
0.015 (0.009-0.026) n=5/5 Case 5 – No Diet (2-11 yo)	
0.050 (0.014-0.157) n=90/90 Case 5 – Diet (2-11 yo)	
**Pantothenic acid**	
0.091 (0.016-0.234) n=9/9 Case 1 (7 do-9 mo) [MMUT]	
0.129 (0.024-0.234) n=3/3 Case 1 – No Diet (7 do-4 mo)	

0.072 (0.016-0.160) n=6/6 Case 1 – Diet (19 do-9 mo)	
0.061 (0.023-0.173) n=38/38 Case 2 (6 do-1½ yo) [MMUT]	
0.085 (0.043-0.140) n=4/4 Case 2 – No Diet (11 do-1 mo)	
0.058 (0.023-0.173) n=34/34 Case 2 – Diet (3 mo-1½ yo)	
0.041 (0.009-0.070) n=11/11 Case 3 (2½-3½ yo) [MMUT]	
0.032 (0.015-0.052) n=27/27 Case 4 (4½-5½ yo) [MMAA]	
0.024 (0.015-0.032) n=9/9 Case 4 – No Diet (4y9mo- 4y11mo)	
0.037 (0.024-0.052) n=18/18 Case 4 – Diet (5yo-5½ yo)	
0.082 (0.014-0.380) n=95/95 Case 5 (2-11 yo) [MMAA]	
0.022 (0.016-0.026) n=5/5 Case 5 – No Diet (2-11 yo)	
0.086 (0.014-0.380) n=90/90 Case 5 – Diet (2-11 yo)	
**Acetone**	
0.351 (0.010-1.788) n=9/9 Case 1 (7 do-9 mo) [MMUT]	
0.032 (0.019-0.051) n=3/3 Case 1 – No Diet (7 do-4 mo)	
0.511 (0.010-1.788) n=6/6 Case 1 – Diet (19 do-9 mo)	
0.024 (0.007-0.084) n=38/38 Case 2 (6 do-1½ yo) [MMUT]	
0.039 (0.015-0.070) n=4/4 Case 2 – No Diet (11 do-1 mo)	
0.022 (0.007-0.084) n=34/34 Case 2 – Diet (3 mo-1½ yo)	
0.037 (0.008-0.260) n=11/11 Case 3 (2½-3½ yo) [MMUT]	
0.016 (0.006-0.091) n=27/27 Case 4 (4½-5½ yo) [MMAA]	
0.023 (0.009-0.091) n=9/9 Case 4 – No Diet (4y9mo- 4y11mo)	
0.013 (0.006-0.023) n=18/18 Case 4 – Diet (5yo-5½ yo)	
0.010 (0.003-0.041) n=95/95 Case 5 (2-11 yo) [MMAA]	
0.010 (0.008-0.013) n=5/5 Case 5 – No Diet (2-11 yo)	
0.010 (0.003-0.041) n=90/90 Case 5 – Diet (2-11 yo)	
**Methylmalonic acid (MMAc)**	
22.349 (6.738-43.860) n=9/9 Case 1 (7 do-9 mo) [MMUT]	
27.317 (19.660-42.150) n=3/3 Case 1 – No Diet (7 do-4 mo)	
19.866 (6.738-43.860) n=6/6 Case 1 – Diet (19 do-9 mo)	
23.084 (8.872-48.500) n=38/38 Case 2 (6 do-1½ yo) [MMUT]	
16.608 (12.780-19.610) n=4/4 Case 2 – No Diet (11 do-1 mo)	
23.846 (8.872-48.500) n=34/34 Case 2 – Diet (3 mo-1½ yo)	
22.848 (6.542-36.580) n=11/11 Case 3 (2½-3½ yo) [MMUT]	0.012±0.017 (1 do) [53].
2.989 (0.174-18.960) n=27/27 Case 4 (4½-5½ yo) [MMAA]	
7.352 (1.607-18.960) n=9/9 Case 4 – No Diet (4y9mo- 4y11mo)	
0.807 (0.174-3.437) n=18/18 Case 4 – Diet (5yo-5½ yo)	
4.085 (0.201-22.070) n=95 Case 5 (2-11 yo) [MMAA]	
15.253 (8.077-22.070) n=5/5 Case 5 – No Diet (2-11 yo)	
3.464 (0.201-9.079) n=90/90 Case 5 – Diet (2-11 yo)	
**Orotic acid (Orot)**	
0.029 (0.007-0.066) n=18/95 Case 5 (2-11 yo) [MMAA]	
0.066 (0.066-0.066) n=1/5 Case 5 – No Diet (2-11 yo)	
0.027 (0.007-0.061) n=17/90 Case 5 – Diet (2-11 yo)	
**Orotidine (Orotid)**	
0.082 (0.035-0.047) n=19/95 Case 5 (2-11 yo) [MMAA]	
0.097 (0.097-0.097) n=1/5 Case 5 – No Diet (2-11 yo)	
0.081 (0.035-0.132) n=18/90 Case 5 – Diet (2-11 yo)	
**L-Carnitine (Car)**	
0.247 (0.147-0.333) n=6/9 Case 1 (7 do-9 mo) [MMUT]	
0.0 n=0/3 Case 1– No Diet (7 do-4 mo)	0.009 ± 0.007 (1 do) [53].
0.247 (0.147-0.333) n=6/6 Case 1 – Diet (19 do-9 mo)	

0.538 (0.156-1.334) n=34/38 (6 do-1½ yo) [MMUT]	
0.0 n=0/4 Case 2 – No Diet (11 do-1 mo)	
0.538 (0.156-1.334) n=34/34 Case 2 – Diet (3 mo-1½ yo)	
0.218 (0.096-0.456) n=5/11 Case 3 (2½-3½ yo) [MMUT]	
0.299 (0.068-0.782) n=17/27 Case 4 (4½-5½ yo) [MMAA]	
0.0 n=0/9 Case 4 – No Diet (4y9mo- 4y11mo)	
0.299 (0.068-0.782) n=17/18 Case 4 – Diet (5yo-5½ yo)	
0.221 (0.024-0.667) n=13/28 Case 5 (2-11 yo) [MMAA]	
0.0 n=1/5 Case 5 – No Diet (2-11 yo)	
0.221 (0.024-0.667) n=13/23 Case 5 – Diet (2-11 yo)	
**Propionylcarnitine (PrCar)**	
0.715 (0.156-1.390) n=6/9 Case 1 (7 do-9 mo) [MMUT]	
0.0 n=0/3 Case 1– No Diet (7 do-4 mo)	
0.715 (0.156-1.390) n=6/6 Case 1 – Diet (19 do-9 mo)	
0.003 (0.001-0.008) n=34/38 Case 2 (6 do-1½ yo) [MMUT]	
0.0 n=0/4 Case 2– No Diet (11 do-1 mo)	
0.922 (0.253-2.266) n=34/34 Case 2 – Diet (3 mo-1½ yo)	
0.361 (0.182-0.612) n=5/11 Case 3 (2½-3½ yo) [MMUT]	<1.20 [8];<br>0.002 (1 do) [53].
0.099 (0.036-0.245) n=17/27 Case 4 (4½-5½ yo) [MMAA]	
0.0 n=0/9 Case 4 – No Diet (4y9mo- 4y11mo)	
0.099 (0.036-0.245) n=17/18 Case 4 – Diet (5yo-5½ yo)	
0.128 (0.044-0.199) n=11/28 Case 5 (2-11 yo) [MMAA]	
0.0 n=0/5 Case 5 – NoDiet (2-11 yo)	
0.128 (0.044-0.199) n=11/23 Case 5 – Diet (2-11 yo)	

[1] For situation when the number of reported samples is smaller than the total number of samples (n=Y/X with Y<X) it means that there were X-Y situations for which the metabolite was not detected (i.e. it was either zero or below the limit of detection).

Table S2. Averaged relative concentrations (mmol/mol Crn) and ranges for individual values (in brackets) for presently monitored MMA patients and previously reported controls. The number of samples n[1] in which a particular metabolite is detected may be lower than the total number of samples investigated.

Averaged Rlative Concentrations and Ranges for MMA Patients (mmol/mol Crn)	Concentrations and Ranges for Controls Previously Reported (mmol/mol Crn)
**Valine (Val)**	
32 (13-83) n=9/9 Case 1 (7 do-9 mo) [MMUT]	11.58 (8.51-15.04) (24-59 yo) [15];<br>6.75 (1.0-18.4) (1 do), 8.26 (5.39-12.07) (25-67 yo) [53];<br>3.4 (2.0-7.7) (19-67 yo) [58];<br>3-26 (0-1 mo), 4-19 (1-6 mo), 6-19 (6-12 mo), 7-21 (1-2 yo), 6-20 (2-4 yo), 3-15 (4-7 yo), 3-17 (7-13 yo), 3-13 (>13 yo) [8];<br>4-28 (0-8 yo), 2-12 (9-18 yo) [51].
29 (13-50) n=3/3 Case 1 – No Diet (7 do-4 mo)	
34 (19-83) n=6/6 Case 1 – Diet (19 do-9 mo)	
20 (3-140) n=35/38 Case 2 (6 do-1½ yo) [MMUT]	
72 (30-140) n=4/4 Case 2 – No Diet (11 do-1 mo)	
14 (3-22) n=31/34 Case 2 – Diet (3 mo-1½ yo)	
7 (3-14) n=9/11 Case 3 (2½-3½ yo) [MMUT]	
5.1 (2-13) n=17/27 Case 4 (4½-5½ yo) [MMAA]	
7 (5-13) n=9/9 Case 4 – No Diet (4y9mo- 4y11mo)	
3 (2-5) n=8/18 Case 4 – Diet (5yo-5½ yo)	
3 (2-8) n=70/95 Case 5 (2-11 yo) [MMAA]	
3 (3-3) n=1/5 Case 5 – No Diet (2-11 yo)	
3 (2-8) n=69/90 Case 5 – Diet (2-11 yo)	
**Lactic acid (Lac)**	
590 (100-2600) n=9/9 Case 1 (7 do-9 mo) [MMUT]	65.78 (21.37-249.66) (24-59 yo)

277 (200-430) n=3/3 Case 1 – No Diet (7 do-4 mo)<br>*747 (100-2600) n=6/6 Case 1 – Diet (19 do-9 mo)*<br>245 (52-2400) n=36/38 Case 2 (6 do-1½ yo) [MMUT]<br>*1290 (210-2400) n=4/4 Case 2 – No Diet (11 do-1 mo)*<br>*114 (52-350) n=32/34 Case 2 – Diet (3 mo-1½ yo)*<br>98 (53-240) n=8/11 Case 3 (2½-3½ yo) [MMUT]<br>98 (49-170) n=3/27 Case 4 (4½-5½ yo) [MMAA]<br>*98 (49-170) n=3/9 Case 4 – No Diet (4y9mo- 4y11mo)*<br>*0 n=0/18 Case 4 – Diet (5yo-5½ yo)*<br>53 (49-60) n=6/95 Case 5 (2-11 yo) [MMAA]<br>*53 (51-55) n=2/5 Case 5 – No Diet (2-11 yo)*<br>*53 (49-60) n=4/90 Case 5 – Diet (2-11 yo)*	[15]; 39.83 (18.37-60.40) (25-67 yo) [14]; 11.6 (3.5-29.3) (19-67 yo) [58]; 166.9 (47-774.2) (1 do) [53]; M/F 28.0 (19.2-35.5)/ 18.84 (13.79-22.30) (1-6 mo), 25.0 (16.4-32.9)/ 28.02 (22.80-35.82) (7-12 mo), 19.6 (17.8-27.2)/ 24.84 (16.21-38.44) (1-2 yo), 21.2 (18.7-24.8)/ 20.4 (14.0-26.0) (2-3 yo) [52]; 0 (0-4 mo), <200 (4-24 mo), <85 (2-10 yo), <50 (>10 yo) [8].
**Alanine (Ala)**	
209 (55-390) n=9/9 Case 1 (7 do-9 mo) [MMUT]<br>*150 (120-200) n=3/3 Case 1 – No Diet (7 do-4 mo)*<br>*239 (55-390) n=6/6 Case 1 – Diet (19 do-9 mo)*<br>168 (77-610) n=38/38 Case 2 (6 do-1½ yo) [MMUT]<br>*428 (290-610) n=4/4 Case 2 – No Diet (11 do-1 mo)*<br>*138 (77-220) n=34/34 Case 2 – Diet (3 mo-1½ yo)*<br>86 (24-260) n=11/11 Case 3 (2½-3½ yo) [MMUT]<br>53 (17-93) n=27/27 Case 4 (4½-5½ yo) [MMAA]<br>*59 (17-93) n=9/9 Case 4 – No Diet (4y9mo- 4y11mo)*<br>*50 (21-90) n=18/18 Case 4 – Diet (5yo-5½ yo)*<br>29 (11-69) n=91/95 Case 5 (2-11 yo) [MMAA]<br>*16 (14-18) n=4/5 Case 5 – No Diet (2-11 yo)*<br>*30 (11-69) n=87/90 Case 5 – Diet (2-11 yo)*	52.38 (14.39-136.02) (24-59 yo) [15]; 45.74 (18.38-91.01) (25-67 yo) [14]; 21.8 (7.1-43.0) (19-67 yo) [58]; 76.6 (32.2-147.8) (1 do) [53]; 75-244 (0-1 mo), 72-206 (1-6 mo), 36-162 (6-12 mo), 41-130 (1-2 yo), 33-115 (2-4 yo), 27-92 (4-7 yo), 17-65 (7-13 yo), 16-68 (>13 yo) [8]; 68-334 (0-1 yo), 14-113 (2-18 yo) [51].
**Pyruvic acid (Pyr)**	
181 (31-690) n=8/9 Case 1 (7 do-9 mo) [MMUT]<br>*89 (37-120) n=3/3 Case 1 – No Diet (7 do-4 mo)*<br>*236 (31-690) n=5/6 Case 1 – Diet (19 do-9 mo)*<br>48 (14-380) n=38/38 Case 2 (6 do-1½ yo) [MMUT]<br>*204 (63-380) n=4/4 Case 2 – No Diet (11 do-1 mo)*<br>*30 (14-78) n=34/34 Case 2 – Diet (3 mo-1½ yo)*<br>17 (10-38) n=8/11 Case 3 (2½-3½ yo) [MMUT]<br>21.4 (10-130) n=20/27 Case 4 (4½-5½ yo) [MMAA]<br>*34 (11-130) n=8/9 Case 4 – No Diet (4y9mo- 4y11mo)*<br>*13 (10-18) n=12/18 Case 4 – Diet (5yo-5½ yo)*<br>12 (9-19) n=59/95 Case 5 (2-11 yo) [MMAA]<br>*10 n=1/5 Case 5 – No Diet (2-11 yo)*<br>*12 (9-19) n=58/90 Case 5 – Diet (2-11 yo)*	38.50 (18.32-69.64) (24-59 yo) [15]; 42.93 (19.73-94.24) (25-67 yo) [14]; 2.7 (0.8-6.4) (19-67 yo) [58]; 36.9 (19.6-89.1) (1 do) [53]; M/F 25.0 (16.9-30.6)/ 18.0 (14.0-23.6) (1-6 mo), 19.0 (14.0-28.0)/ 29.1 (17.3-35.1) (7-12 mo), 25.1 (18.0-31.1)/ 30.3 (12.1-40.0) (1-2 yo), 22.5 (15.6-27.6)/ 24.9 (19.0-30.7) (2-3 yo) [52]; 0-22 [8].
**Citric acid (Cit)**	
457 (44-1100) n=9/9 Case 1 (7 do-9 mo) [MMUT]<br>*317 (100-730) n=3/3 Case 1 Case 1 – No Diet (7 do-4 mo)]*<br>*527 (44-1100) n=6/6 Case 1 – Diet (19 do-9 mo)*<br>511 (68-1100) n=38/38 Case 2 (6 do-1½ yo) [MMUT]<br>*400 (170-640) n=4/4 Case 2 – No Diet (11 do-1 mo)*<br>*524 (68-1100) n=34/34 Case 2 – Diet (3 mo-1½ yo)*<br>422 (52-1300) n=10/11 Case 3 (2½ - 3½ yo) [MMUT]<br>264 (45-460) n=25/27 Case 4 (4½-5½ yo) [MMAA]<br>*200 (45-340) n=7/9 Case 4– No Diet (4y9mo- 4y11mo)*	242.34 (81.79-403.84) (24-59 yo) [15]; 235.55 (45.81-394.38) (25-67 yo) [14]; 203 (49-600) (19-67 yo) [58]; 396.6 (39.6-893.5) (1 do) [53].

289 (190-460) n=18/18 Case 4 – Diet (5yo-5½ yo)	
94 (41-270) n=23/95 Case 5 (2-11 yo) [MMAA]	
0 n=0/5 Case 5 – No Diet (2-11 yo)	
94 (41-270) n=23/90 Case 5– Diet (2-11 yo)	
**Dimethylamine (DMA)**	
110 (85-130) n=8/9 Case 1 (7 do-9 mo) [MMUT]	30.35 (16.30-50.36) (24-59 yo) [15];<br>28.67 (10.26-57.81) (25-67 yo) [14];<br>30.8 (20.3-59.2) (19-67 yo) [58].
103 (85-120) n=2/3 Case 1 – No Diet (7 do-4 mo)]	
113 (90-130) n=6/6 Case 1 – Diet (19 do-9 mo)	
109 (42-180) n=38/38 Case 2 (6 do-1½ yo) [MMUT]	
128 (42-180) n=4/4 Case 2 – No Diet (11 do-1 mo)	
107 (95-130) n=34/34 Case 2 – Diet (3 mo-1½ yo)	
94 (81-1200) n=11/11 Case 3 (2½-3½ yo) [MMUT]	
78 (50-110) n=27/27 Case 4 (4½-5½ yo) [MMAA]	
72 (50-83) n=9/9 Case 4 – No Diet (4y9mo- 4y11mo)	
81 (68-110) n=18/18 Case 4 – Diet (5yo-5½ yo)	
62 (32-110) n=95/95 Case 5 (2-11 yo) [MMAA]	
43 (34-51) n=5/5 Case 5 – No Diet (2-11 yo)	
63 (32-110) n=90/90 Case 5 – Diet (2-11 yo)	
**Trimethylamine (TMA)**	
4 (3-6) n=9/9 Case 1 (7 do-9 mo) [MMUT]	2.5 (0.3-19.4) (19-67 yo) [58];<br><5 (<1 wo) [S1].
5 (4-6) n=3/3 Case 1 – No Diet (7 do-4 mo)]	
4 (3-4) n=6/6 Case 1 – Diet (19 do-9 mo)	
12 (2-170) n=28/38 Case 2 (6 do-1½ yo) [MMUT]	
4 (3-6) n=4/4 Case 2 – No Diet (11 do-1 mo)	
14 (2-170) n=24/34 Case 2 – Diet (3 mo-1½ yo)	
6 (3-8) n=3/11 Case 3 (2½-3½ yo) [MMUT]	
4 (2-10) n=6/27 Case 4 (4½-5½ yo) [MMAA]	
2 (2-2) n=2/9 Case 4 – No Diet (4y9mo- 4y11mo)	
5 (2-10) n=4/18 Case 4 – Diet (5yo-5½ yo)	
7 (2-21) n=18/95 Case 5 (2-11 yo) [MMAA]	
0 n=0/5 Case 5 – No Diet (2-11 yo)	
7 (2-21) n=18/90 Case 5 – Diet (2-11 yo)	
**Glycine (Gly)**	
2322 (120-7300) n=9/9 Case 1 (7 do-9 mo) [MMUT]	106 (44-300) (19-67 yo) [58];<br>362.1 (102.6-853.6) (1 do) [53];<br>283-1097 (0-1 mo), 210-743 (1-6 mo), 114-445 (6-12 mo), 110-356 (1-2 yo), 11-326 (2-4 yo), 91-246 (4-7 yo), 64-236 (7-13 yo), 43-173 (>13 yo) [8];<br>113-1427 (0-1 mo), 147-570 (2-12 mo), 66-453 (1-8 yo), 51-238 (9-18 yo) [51].
833 (250-1300) n=3/3 Case 1 – No Diet (7 do-4 mo)	
3067 (120-7300) n=6/6 Case 1 – Diet (19 do-9 mo)	
2277 (680-4400) n=38/38 Case 2 (6 do-1½ yo) [MMUT]	
3100 (2400-3900) n=4/4 Case 2 – No Diet (11 do-1 mo)	
2180 (680-4400) n=34/34 Case 2 – Diet (3 mo-1½ yo)	
602 (140-1400) n=11/11 Case 3 (2½-3½ yo) [MMUT]	
148 (69-330) n=27/27 Case 4 (4½-5½ yo) [MMAA]	
211 (110-330) n=9/9 Case 4 – No Diet (4y9mo- 4y11mo)	
117 (69-240) n=18/18 Case 4 – Diet (5yo-5½ yo)	
66 (34-240) n=79/95 Case 5 (2-11 yo) [MMAA]	
41 (38-46) n=3/5 Case 5 – No Diet (2-11 yo)	
67 (34-240) n=76/90 Case 5 – Diet (2-11 yo)	
**Hippuric acid (Hip)**	
900 (900-900) n=1/9 Case 1 (7 do-9 mo) [MMUT]	189.34 (60.44-521.82) (24-59 yo) [15];<br>246.76 (37.40-766.16) (25-67 yo)<br>28.67 (10.26-57.81) (25-67 yo)
0 n=0/3 Case 1 – No Diet (7 do-4 mo)	
900 n=1/6 Case 1 – Diet (19 do-9 mo)	
570 (200-2000) n=6/38 Case 2 (6 do-1½ yo) [MMUT]	

200 n=1/4 Case 2 – No Diet (11 do-1 mo)<br>*644 (200-2000) n=5/34 Case 2 – Diet (3 mo-1½ yo)*<br>640 (320-960) n=2/11 Case 3 (2½-3½ yo) [MMUT]<br>442 (170-1200) n=21/27 Case 4 (4½-5½ yo) [MMAA]<br>*389 (170-710) n=7/9 Case 4– No Diet (4y9mo- 4y11mo)*<br>*469 (170-1200) n=14/18 Case 4– Diet (5yo-5½ yo)*<br>443 (190-1300) n=39/95 Case 5 (2-11 yo) [MMAA]<br>*265 (190-340) n=2/5 Case 5 – No Diet (2-11 yo)*<br>*452 (200-1300) n=37/90 Case 5 – Diet (2-11 yo)*	[14];<br>229 (19-622) (19-67 yo) [58];<br>64.1 (1.51-213.0) (1 do) [53];<br>M/F 24.3 (12.5-56.2)/ 75.0 (31.7-157.4) (1-6 mo), 33.8 (19.0-63.0)/ 57.3 (13.5-131.5) (7-12 mo), 79.9 (45.1-173.8)/ 40.6 (14.0-103.0) (1-2 yo), 87.7 (51.0-163.0)/ 62.6 (31.5-121.5) (2-3 yo) [52].
**Formic acid (For)**	
69 (16-200) n=9/9 Case 1 (7 do-9 mo) [MMUT]<br>*76 (60-99) n=3/3 Case 1 – No Diet (7 do-4 mo)*<br>*65 (16-200) n=6/6 Case 1 – Diet (19 do-9 mo)*<br>52 (11-400) n=38/38 Case 2 (6 do-1½ yo) [MMUT]<br>*175 (75-400) n=4/4 Case 2 – No Diet (11 do-1 mo)*<br>*37 (11-100) n=34/34 Case 2 – Diet (3 mo-1½ yo)*<br>640 (320-960) n=7/11 Case 3 (2½-3½ yo) [MMUT]<br>18 (12-28) n=19/27 Case 4 (4½-5½ yo) [MMAA]<br>*15 (12-19) n=3/9 Case 4 – No Diet (4y9mo- 4y11mo)*<br>*19 (12-28) n=16/18 Case 4 – Diet (5yo-5½ yo)*<br>18 (10-49) n=57/95 Case 5 (2-11 yo) [MMAA]<br>*13 (12-14) n=2/5 Case 5 – No Diet (2-11 yo)*<br>*18 (10-49) n=55/90 Case 5 – Diet (2-11 yo)*	55.42 (14.60-120.70) (24-59 yo) [15];<br>32.31 (10.55-78.90) (25-67 yo) [14];<br>26.8 (6.9-120.9) (19-67 yo) [58];<br>160 (30-740) (1-7 do) [24];<br>290 (<340) (<1 wo) [S1].
**1-Methylguanidine**	
133 (93-170) n=9/9 Case 1 (7 do-9 mo) [MMUT]<br>*153 (140-170) n=3/3 Case 1 – No Diet (7 do-4 mo)*<br>*122 (93-140) n=6/6 Case 1 – Diet (19 do-9 mo)*<br>128 (64-360) n=38/38 Case 2 (6 do-1½ yo) [MMUT]<br>*240 (170-360) n=4/4 Case 2 – No Diet (11 do-1 mo)*<br>*114 (64-290) n=34/34 Case 2 – Diet (3 mo-1½ yo)*<br>165 (69-510) n=7/11 Case 3 (2½-3½ yo) [MMUT]<br>80 (64-110) n=19/27 Case 4 (4½-5½ yo) [MMAA]<br>*81 (69-110) n=9/9 Case 4 – No Diet (4y9mo- 4y11mo)*<br>*78 (64-110) n=18/18 Case 4 – Diet (5yo-5½ yo)*<br>72 (64-98) n=33/95 Case 5 (2-11 yo) [MMAA]<br>*0 n=0/5 Case 5 – No Diet (2-11 yo)*<br>*72 (64-98) n=33/90 Case 5 – Diet (2-11 yo)*	2.7 (1.2-6.0) (19-67 yo) [58];<br>160 (<1 wo) [S1].
**Betaine**	
1017 (170-840) n=7/9 Case 1 (7 do-9 mo) [MMUT]<br>*717 (630-200) n=3/3 Case 1 – No Diet (7 do-4 mo)*<br>*1243 (170-1900) n=4/6 Case 1 – Diet (19 do-9 mo)*<br>207 (38-1500) n=38/38 Case 2 (6 do-1½ yo) [MMUT]<br>*810 (48-1500) n=4/4 Case 2 – No Diet (11 do-1 mo)*<br>*136 (38-480) n=34/34 Case 2 – Diet (3 mo-1½ yo)*<br>107 (41-220) n=11/11 Case 3 (2½-3½ yo) [MMUT]<br>35 (9-100) n=26/27 Case 4 (4½-5½ yo) [MMAA]<br>*22 (10-30) n=9/9 Case 4 – No Diet (4y9mo- 4y11mo)*<br>*41 (9-100) n=17/18 Case 4 – Diet (5yo-5½ yo)*<br>42 (11-130) n=94/95 Case 5 (2-11 yo) [MMAA]<br>*18 (15-23) n=5/5 Case 5 – No Diet (2-11 yo)*<br>*43 (11-130) n=89/90 Case 5 – Diet (2-11 yo)*	11.5 (2.7-24.7) (19-67 yo) [58];<br>189.6 (74.1-410.3) (1 do) [53].

**Creatine (Crea)**	
417 (67-1200) n=8/9 Case 1 (7 do-9 mo) [MMUT]	12-682 (<4 yo), 29-1551 (<10 yo), 19-1046 (10-15 yo), 7-492 (>15 yo) [8]; 46 (3-448) (19-67 yo) [58]; 56.0 (4.66-455.3) (1 do) [53]; 210.15 (25-776) (21 do) [59]; 6-1208 (<4 yo), 17-720 (4-12 yo), 11-244 (>12 yo) [S2].
199 (68-430) n=3/3 Case 1 – No Diet (7 do-4 mo)	
547 (67-1200) n=5/6 Case 1 – Diet (19 do-9 mo)	
1007 (340-2100) n=38/38 Case 2 (6 do-1½ yo) [MMUT]	
1258 (880-2100) n=4/4 Case 2 – No Diet (11 do-1 mo)	
977 (340-1800) n=34/34 Case 2 – Diet (3 mo-1½ yo)	
348 (84-710) n=10/11 Case 3 (2½-3½ yo) [MMUT]	
264 (140-390) n=7/27 Case 4 (4½-5½ yo) [MMAA]	
252 (140-390) n=6/9 Case 4 – No Diet (4y9mo- 4y11mo)	
340 n=1/18 Case 4 – Diet (5yo-5½ yo)	
211 (56-660) n=49/95 Case 5 (2-11 yo) [MMAA]	
62 (56-67) n=2/5 Case 5 – No Diet (2-11 yo)	
217 (58-660) n=47/90 Case 5 – Diet (2-11 yo)	
***N,N*-Dimethylglycine**	
75 (22-150) n=9/9 Case 1 (7 do-9 mo) [MMUT]	4.4 (1.6-10.4) (19-67 yo) [58]; 80 (30-270) (< 7 do) [24].
78 (27-150) n=3/3 Case 1 – No Diet (7 do-4 mo)	
73 (22-140) n=6/6 Case 1 – Diet (19 do-9 mo)	
23 (11-100) n=38/38 Case 2 (6 do-1½ yo) [MMUT]	
77 (56-100) n=4/4 Case 2 – No Diet (11 do-1 mo)	
17 (11-30) n=34/34 Case 2 – Diet (3 mo-1½ yo)	
16 (11-30) n=11/11 Case 3 (2½-3½ yo) [MMUT]	
19 (5-50) n=27/27 Case 4 (4½-5½ yo) [MMAA]	
14 (5-27) n=9/9 Case 4 – No Diet (4y9mo- 4y11mo)	
22 (10-50) n=18/18 Case 4 – Diet (5yo-5½ yo)	
14 (5-36) n=93/95 Case 5 (2-11 yo) [MMAA]	
9 (8-9) n=5/5 Case 5 – No Diet (2-11 yo)	
14 (5-36) n=88/90 Case 5 – Diet (2-11 yo)	
**Taurine**	
1606 (300-4600) n=7/9 Case 1 (7 do-9 mo) [MMUT]	8-226 (0-1 mo), 6-89 (1-6 mo), 9-123 (6-12 mo), 12-159 (1-2 yo), 13-200 (2-4 yo), 17-230 (4-7 yo), 18-230 (7-13 yo), 16-180 (>13 yo) [8]; 81 (13-251) (19-67 yo) [58]; 1067 (74.6-2841) (1 do) [53].
940 n=1/3 Case 1 – No Diet (7 do-4 mo)	
1717 (300-4600) n=6/6 Case 1 – Diet (19 do-9 mo)	
586 (190-3300) n=28/38 Case 2 (6 do-1½ yo) [MMUT]	
967 (200-1600) n=3/4 Case 2 – No Diet (11 do-1 mo)	
540 (190-3300) n=25/34 Case 2 – Diet (3 mo-1½ yo)	
1014 (140-2300) n=9/11 Case 3 (2½-3½ yo) [MMUT]	
191 (140-290) n=12/27 Case 4 (4½-5½ yo) [MMAA]	
0 n=0/9 Case 4 – No Diet (4y9mo- 4y11mo)	
191 (140-290) n=12/18 Case 4 – Diet (5yo-5½ yo)	
907 (150-3700) n=34/95 Case 5 (2-11 yo) [MMAA]	
0 n=0/5 Case 5– No Diet (2-11 yo)	
907 (150-3700) n=34/90 Case 5 – Diet (2-11 yo)	
**Acetic acid**	
51 (26-85) n=9/9 Case 1 (7 do-9 mo) [MMUT]	13.0 (2.5-106.0) (19-67 yo) [58].
46 (37-61) n=3/3 Case 1 – No Diet (7 do-4 mo)	
54 (26-85) n=6/6 Case 1 – Diet (19 do-9 mo)	
55 (26-200) n=38/38 Case 2 (6 do-1½ yo) [MMUT]	
132 (68-200) n=4/4 Case 2 – No Diet (11 do-1 mo)	
46 (26-72) n=34/34 Case 2 – Diet (3 mo-1½ yo)	
25 (11-62) n=11/11 Case 3 (2½-3½ yo) [MMUT]	
15 (7-37) n=26/27 Case 4 (4½-5½ yo) [MMAA]	

15 (7-37) n=9/9 Case 4 – No Diet (4y9mo- 4y11mo)<br>*15 (9-29) n=17/18 Case 4 – Diet (5yo-5½ yo)*<br>19 (5-53) n=95/95 Case 5 (2-11 yo) [MMAA]<br>*14 (9-18) n=5/5 Case 5 – No Diet (2-11 yo)*<br>*19 (5-53) n=90/90 Case 5 – Diet (2-11 yo)*	
**Fumaric acid**	
22 (7-53) n=9/9 Case 1 (7 do-9 mo) [MMUT]<br>*12 (7-17) n=3/3 Case 1 – No Diet (7 do-4 mo)*<br>*27 (12-53) n=6/6 Case 1 – Diet (19 do-9 mo)*<br>14 (5-120) n=38/38 Case 2 (6 do-1½ yo) [MMUT]<br>*56 (23-120) n=4/4 Case 2 – No Diet (11 do-1 mo)*<br>*9 (5-16) n=34/34 Case 2 – Diet (3 mo-1½ yo)*<br>8 (3-21) n=11/11 Case 3 (2½-3½ yo) [MMUT]<br>6 (2-34) n=19/27 Case 4 (4½-5½ yo) [MMAA]<br>*9 (3-34) n=9/9 Case 4 – No Diet (4y9mo- 4y11mo)*<br>*3 (2-4) n=10/18 Case 4 – Diet (5yo-5½ yo)*<br>3 (2-5) n=14/95 Case 5 (2-11 yo) [MMAA]<br>*0 n=0/5 Case 5 – No Diet (2-11 yo)*<br>*3 (2-5) n=14/90 Case 5 – Diet (2-11 yo)*	0.7 (0.2-1.7) (19-67 yo) [58];<br>14.2 (2.09-37.7) (1 do) [53];<br>M/F 3 (0.15-5.69)/ 1.0 (0.0-1.5) (1-6 mo), 1.52 (0.0-2.0)/ 2.4 (1.2-4.3) (7-12 mo), 1.70 (0.12-2.5)/ 1.2 (0.0-2.6) (1-2 yo), 0.6 (0.0-2.0)/ 1.6 (0.0-2.9) (2-3 yo) [52].
**Succinic acid**	
96 (7-210) n=9/9 Case 1 (7 do-9 mo) [MMUT]<br>*71 (57-87) n=3/3 Case 1 – No Diet (7 do-4 mo)*<br>*109 (7-210) n=6/6 Case 1 – Diet (19 do-9 mo)*<br>73 (21-210) n=38/38 Case 2 (6 do-1½ yo) [MMUT]<br>*134 (53-210) n=4/4 Case 2 – No Diet (11 do-1 mo)*<br>*65 (21-140) n=34/34 Case 2 – Diet (3 mo-1½ yo)*<br>43 (21-97) n=11/11 Case 3 (2½-3½ yo) [MMUT]<br>48 (15-89) n=27/27 Case 4 (4½-5½ yo) [MMAA]<br>*24 (15-41) n=9/9 Case 4 – No Diet (4y9mo- 4y11mo)*<br>*59 (22-89) n=18/18 Case 4 – Diet (5yo-5½ yo)*<br>16 (5-72) n=93/95 Case 5 (2-11 yo) [MMAA]<br>*6 (5-8) n=3/5 Case 5 – No Diet (2-11 yo)*<br>*16 (5-72) n=90/90 Case 5 – Diet (2-11 yo)*	40-125 (0-4 mo), 44-79 (4-24 mo), 5-81 (2-10 yo), <16 (>10 yo) [8];<br>6.0 (0.3-33.3) (19-67 yo) [58];<br>21.8 (4.35-81.2) (1 do) [53];<br>M/F 58.4 (15.7-69.5)/ 17.0 (11.9-24.1) (1-6 mo), 34.0 (22.0-42.0)/ 28.5 (20.2-49.6) (7-12 mo), 29.0 (19.8-38.0)/ 23.3 (15.0-33.0) (1-2 yo), 19.0 (14.0-27.0)/ 20.2 (14.0-34.4) (2-3 yo) [52].
**Pantothenic acid**	
60 (21-120) n=9/9 Case 1 (7 do-9 mo) [MMUT]<br>*74 (21-100) n=3/3 Case 1 – No Diet (7 do-4 mo)*<br>*53 (26-120) n=6/6 Case 1 – Diet (19 do-9 mo)*<br>42 (20-170) n=38/38 Case 2 (6 do-1½ yo) [MMUT]<br>*99 (49-170) n=4/4 Case 2 – No Diet (11 do-1 mo)*<br>*35 (20-91) n=34/34 Case 2 – Diet (3 mo-1½ yo)*<br>19 (17-24) n=8/11 Case 3 (2½-3½ yo) [MMUT]<br>19 (17-21) n=3/27 Case 4 (4½-5½ yo) [MMAA]<br>*19 (17-21) n=2/9 Case 4 – No Diet (4y9mo- 4y11mo)*<br>*18 n=1/18 Case 4 – Diet (5yo-5½ yo)*<br>54 (16-130) n=38/95 Case 5 (2-11 yo) [MMAA]<br>*0 n=0/5 Case 5 – No Diet (2-11 yo)*<br>*54 (16-130) n=38/90 Case 5 – Diet (2-11 yo)*	1.9 (0.6-4.4) (19-67 yo) [58].
**Acetone**	
245 (11-1400) n=9/9 Case 1 (7 do-9 mo) [MMUT]<br>*19 (17-22) n=3/3 Case 1 – No Diet (7 do-4 mo)*<br>*358 (11-1400) n=6/6 Case 1 – Diet (19 do-9 mo)*	3.9 (0.8-17.6) (19-67 yo) [58];<br>4.2 (0.98-15.3) (<1 yo), 0.92 (0.20-2.8) (>18 yo) [47].

18 (5-93) n=38/38 Case 2 (6 do-1½ yo) [MMUT]<br>*45 (17-90) n=4/4 Case 2 – No Diet (11 do-1 mo)*<br>*15 (5-93) n=34/34 Case 2 – Diet (3 mo-1½ yo)*<br>16 (4-87) n=11/11 Case 3 (2½-3½ yo) [MMUT]<br>8 (3-62) n=27/27 Case 4 (4½-5½ yo) [MMAA]<br>*14 (4-62) n=9/9 Case 4 – No Diet (4y9mo- 4y11mo)*<br>*4 (3-6) n=18/18 Case 4 – Diet (5yo-5½ yo)*<br>3 (2-11) n=89/95 Case 5 (2-11 yo) [MMAA]<br>*4 (2-5) n=5/5 Case 5 – No Diet (2-11 yo)*<br>*3 (2-11) n=84/90 Case 5 – Diet (2-11 yo)*	
**Methylmalonic acid (MMAc)**	
16111 (7000-25000) n=9/9 Case 1 (7 do-9 mo) [MMUT]<br>*17000 (15000-18000) n=3/3 Case 1 – No Diet (7 do-4 mo)*<br>*15667 (7000-25000) n=6/6 Case 1 – Diet (19 do-9 mo)*<br>15350 (5600-28000) n=38/38 Case 2 (6 do-1½ yo) [MMUT]<br>*20000 (14000-28000) n=4/4 Case 2 – No Diet (11 do-1 mo)*<br>*14803 (5600-24000) n=34/34 Case 2 – Diet (3 mo-1½ yo)*<br>11645 (5600-25000) n=11/11 Case 3 (2½-3½ yo) [MMUT]<br>1624 (76-14000) n=27/27 Case 4 (4½-5½ yo) [MMAA]<br>*4369 (760-14000) n=9/9 Case 4 – No Diet (4y9mo- 4y11mo)*<br>*251 (76-690) n=18/18 Case 4 – Diet (5yo-5½ yo)*<br>1259 (130-7900) n=95/95 Case 5 (2-11 yo) [MMAA]<br>*4980 (3200-7900) n=5/5 Case 5 – No Diet (2-11 yo)*<br>*1052 (130-3800) n=90/90 Case 5 – Diet (2-11 yo)*	1-11 (0-4 mo), 2-13 (4-24 mo), 1-4 (2-10 yo), 0-4 (>10 yo) [8];<br>0.58-3.56 [54];<br>1.9 (0.7-3.5) (19-67 yo) [58];<br>2.79 (0.42-19.7) (1 do) [53].
**Orotic acid (Orot)**	
8 (5-16) n=18/95 Case 5 (2-11 yo) [MMAA]<br>*22 n=1/5 Case 5 – No Diet (2-11 yo)*<br>*12 (2-26) n=17/90 Case 5 – Diet (2-11 yo*	1.85 (0-5) (21 do) [59].
**Orotidine (Orotid)**	
35 (21-55) n=19/95 Case 5 (2-11 yo) [MMAA]<br>*32 n=1/5 Case 5 – No Diet (2-11 yo)*<br>*36 (21-55) n=18/90 Case 5 – Diet (2-11 yo)*	
**L-Carnitine (Car)**	
346 (189-838) n=6/9 Case 1 (7 do-9 mo) [MMUT]<br>*0 n=0/3 Case 1 – No Diet (7 do-4 mo)*<br>*346 (189-838) n=6/6 Case 1 – Diet (19 do-9 mo)*<br>520 (162-1028) n=34/38 Case 2 (6 do-1½ yo) [MMUT]<br>*0 n=0/4 Case 2 – No Diet (11 do-1 mo)*<br>*520 (162-1082) n=34/34 Case 2 – Diet (3 mo-1½ yo)*<br>153 (95-226) n=5/11 Case 3 (2½-3½ yo) [MMUT]<br>165 (40-342) n=17/27 Case 4 (4½-5½ yo) [MMAA]<br>*0 n=0/9 Case 4 – No Diet (4y9mo- 4y11mo)*<br>*165 (40-342) n=17/18 Case 4 – Diet (5yo-5½ yo)*<br>109 (15-429) n=13/28 Case 5 (2-11 yo) [MMAA]<br>*0 n=0/5 Case 5 – No Diet (2-11 yo)*<br>*109 (15-429) n=13/23 Case 5 – Diet (2-11 yo)*	5.0 (0.7-16.4) (19-67 yo) [58];<br>2.01 (0.79-5.67) (1 do) [53].
**Propionylcarnitine (PrCar)**	
700 (206-1186) n=6/9 Case 1 (7 do-9 mo) [MMUT]<br>*0 n=0/3 Case 1 – No Diet (7 do-4 mo)*<br>*700 (206-1186) n=6/6 Case 1 – Diet (19 do-9 mo)*<br>902 (404-1624) n=34/38 Case 2 (6 do-1½ yo) [MMUT]	0.07 (0.01-0.20) (19-67 yo) [58];<br>0.03 (0.01-0.07) (1 do) [53].

0 n=0/4 Case 2 – No Diet (11 do-1 mo)
902 (404-1624) n=34/34 Case 2– Diet (3 mo-1½ yo)
262 (187-356) n=5/11 Case 3 (2½-3½ yo) [MMUT]
55 (21-106) n=17/27 Case 4 (4½-5½ yo) [MMAA]
0 n=0/9 Case 4 – No Diet (4y9mo- 4y11mo)
55 (21-106) n=17/18 Case 4 – Diet (5yo-5½ yo)
65 (40-124) n=11/28 Case 5 (2-11 yo) [MMAA]
0 n=0/5 Case 5 – No Diet (2-11 yo)
65 (40-124) n=11/23 Case 5 – Diet (2-11 yo)

[1] For situation when the number of reported samples is smaller than the total number of samples (n=Y/X with Y<X) it means that there were X-Y situations for which the metabolite was not detected (i.e. it was either zero or below the limit of detection).

References

[S1] Embade, N.; Cannet, C.; Diercks, T.; Gil-Redondo, R.; Bruzzone, C.; Ansó, S.; Echevarría, L.R.; Ayucar, M.M.M.; Collazos, L.; Lodoso, B.; Guerra, E.; Elorriaga, I.A.; Kortajarena, M.Á.; Legorburu, A.P.; Fang, F.; Astigarraga, I.; Schäfer, H.; Spraul, M.; Millet, O. NMR-based newborn urine screening for optimized detection of inherited errors of metabolism. *Sci. Rep.* **2019**, *9*, 13067.

[S2] Nouioua, S.; Cheillan, D.; Zaouidi, S.; Salomons, G.S.; Amedjout, N.; Kessaci, F.; Boulahdour, N.; Hamadouche, T.; Tazir, M. Creatine deficiency syndrome. A treatable myopathy due to arginine-glycine amidinotransferase (AGAT) deficiency. *Neuromuscul. Disord.* **2013**, *23*, 670-674.
